# Behavioral Quantification of Audiomotor Transformations in Improvising and Score-Dependent Musicians

**DOI:** 10.1371/journal.pone.0166033

**Published:** 2016-11-11

**Authors:** Robert Harris, Peter van Kranenburg, Bauke M. de Jong

**Affiliations:** 1 Department of Neurology, University Medical Center Groningen, University of Groningen, Groningen, The Netherlands; 2 BCN Neuroimaging Center, University of Groningen, Groningen, The Netherlands; 3 Prince Claus Conservatoire, Hanze University of Applied Sciences, Groningen, The Netherlands; 4 Meertens Institute, Amsterdam, The Netherlands; University of Western Ontario, CANADA

## Abstract

The historically developed practice of learning to play a music instrument from notes instead of by imitation or improvisation makes it possible to contrast two types of skilled musicians characterized not only by dissimilar performance practices, but also disparate methods of audiomotor learning. In a recent fMRI study comparing these two groups of musicians while they either imagined playing along with a recording or covertly assessed the quality of the performance, we observed activation of a right-hemisphere network of posterior superior parietal and dorsal premotor cortices in improvising musicians, indicating more efficient audiomotor transformation. In the present study, we investigated the detailed performance characteristics underlying the ability of both groups of musicians to replicate music on the basis of aural perception alone. Twenty-two classically-trained improvising and score-dependent musicians listened to short, unfamiliar two-part excerpts presented with headphones. They played along or replicated the excerpts by ear on a digital piano, either with or without aural feedback. In addition, they were asked to harmonize or transpose some of the excerpts either to a different key or to the relative minor. MIDI recordings of their performances were compared with recordings of the aural model. Concordance was expressed in an audiomotor alignment score computed with the help of music information retrieval algorithms. Significantly higher alignment scores were found when contrasting groups, voices, and tasks. The present study demonstrates the superior ability of improvising musicians to replicate both the pitch and rhythm of aurally perceived music at the keyboard, not only in the original key, but also in other tonalities. Taken together with the enhanced activation of the right dorsal frontoparietal network found in our previous fMRI study, these results underscore the conclusion that the practice of improvising music can be associated with enhanced audiomotor transformation in response to aurally perceived music.

## Introduction

Classical music offers an interesting window on motor learning, not only because of the high level of motor control exhibited in performance [1], but especially because of the historically developed practice in Western culture of using sheet music not only to learn specific pieces, but also to learn how to play the instrument itself [2]. While in classical music education, great emphasis is placed on aural skills such as the identification of intervals and triads and their inversions, the skill of playing music ‘by ear’ is rarely taught or assessed. Classical musicians are *de facto* ‘score-dependent’, a term which refers not only to the fact that the music performed is an artistic representation of the music score, but also that it is learned from the printed score and not by aural imitation. From a global perspective, however, score-dependence may be considered to be the exception. All over the world, both in the past and in the present, instrumental music was and is generally learned by imitation and improvisation [3], a practice which intuitively seems more compatible with the learning of an audiomotor skill. Surprisingly, however, little study has been made of the relationship of the practice of improvisation with the development of audiomotor integration.

With few exceptions, neuroscientific studies to date have recruited mainly classically-trained musicians [4–13]. Studies contrasting improvising with score-dependent musicians are scarce. Tervaniemi and colleagues [14] noted an advantage for improvising musicians not only in the conscious detection of changes in melodic patterns, but also in subsequent brain responses to these changes during non-attentive listening. These results were corroborated and extended in a study by Vuust and colleagues [15] contrasting non-musicians not only with classical, but also with jazz and rock musicians. In contrast with all other groups, including classical musicians, jazz musicians exhibited significantly higher mean mismatch negativity (MMN) amplitudes to pitch, timbre, intensity, and rhythm. Behavioral scores as measured by AMMA, the Advanced Measures of Musical Audition [16] were not higher for jazz musicians, with the exception of the rhythm subtest. In a behavioral study, however, Woody and Lehmann [17] demonstrated that ‘vernacular’ musicians outperformed ‘formal’ musicians in aural learning, the latter requiring twice the number of trials to achieve accuracy in vocal reproduction of a melody and almost three times as many trials to achieve accuracy in instrumental reproduction of a melody (by ear).

The observed differences may be understood in the context of the procedural-declarative model of learning and memory [18] and the associated dual-stream model of action and perception [19] which propose that online recruitment of implicit, procedural knowledge via the dorsal stream enhances performance without the prerequisite of declarative knowledge [20], a phenomenon which can be observed in children who have clearly mastered the language, but know little about grammar. Musicians who play ‘by ear’ would therefore be able to employ procedural knowledge of music syntax to enhance audiomotor performance without knowing much about music theory or harmony. At the same time, score-dependent musicians who have acquired extensive declarative knowledge of music theory and harmony might not necessarily have acquired comparable *procedural* knowledge of music syntax.

The transformation of imagined or perceived pitch into goal-directed movement while playing a music instrument is a function of parietal cortex, just as the transformation of visually perceived music notation. The involvement of parietal cortex in audiomotor transformations has been demonstrated by imaging studies implicating the superior parietal cortex, in particular the intraparietal sulcus (IPS), not only in music transposition [21] and retrograde musical transformations [22], but also in pitch-to-space transformations [23]. Similar parietal activations have also been observed in pianists while sight-reading music and have been interpreted as reflecting the visuomotor transformation of music notation into spatial keyboard coordinates [24–25, 2]. It is not inconceivable that score-dependent performance practice might bias sensorimotor learning in the direction of *visuomotor* learning, rather than audiomotor learning. The inability to play ‘by ear’ would be a logical consequence.

In a recent fMRI experiment, we assessed cerebral activations in improvising and score-dependent musicians while they imagined playing along with both familiar and unfamiliar excerpts composed in the two-part, tonal style. A crucial difference between the two groups was the significantly larger activation of a right hemisphere network of posterior superior parietal and dorsal premotor areas observed in improvising musicians. This was interpreted as evidence of enhanced pitch-to-keyboard space transformation, pointing towards the superior ability of improvising musicians to perform audiomotor transformations while listening to music [26].

In the present study, we investigated the instrumental performance of both groups of musicians, quantifying their ability to organize playing movements cued by aurally perceived music in an audiomotor alignment score. Our hypothesis was that improvising musicians would exhibit superior ability to replicate and transpose aurally perceived music on their instrument.

## Materials and Methods

This study was approved by the Medical Ethics Committee of the University Medical Center Groningen, Groningen, The Netherlands. All subjects gave written informed consent in accordance with the Declaration of Helsinki (2008), prior to participation.

### Subjects

The improvising and score-dependent musicians who participated in this study had all previously participated in an fMRI study of audiomotor integration [26]. The group of improvising musicians consisted of eleven organists and one pianist while the group of score-dependent musicians consisted of ten pianists. All subjects were male. They were recruited from all over The Netherlands. The distinction between improvising and score-dependent musicians was not based on formal assessment of their ability to improvise, but on the nature of their performance practice i.e. whether or not their professional keyboard performances involved improvisation.

In The Netherlands, the eighteenth-century practice of keyboard improvisation has persisted among church organists. Organists are accustomed to improvise preludes and postludes before and after the service as well as introductions, intermezzos, and modulations while harmonizing and accompanying psalms and hymns. Organ concerts feature improvisation and many organists participate in improvisation competitions. Of the eleven organists, seven had participated in international improvisation competitions and six had won prizes. By contrast, the professional practice of the participating score-dependent pianists involved performance of the repertoire as notated and did not include extemporization. Like most classically-trained musicians, these pianists learn the pieces they perform from the printed score, frequently committing them to memory and performing by heart.

With the exception of two conservatory students (third and fourth year, one from each group), subjects had all completed one or more conservatory degrees in classical music performance in organ or piano. During their professional training, organists received the same instruction in music theory and ear training as score-dependent pianists and performed compositions learned from music notation just as their score-dependent colleagues. As piano is the required secondary instrument for organists in The Netherlands, all organists were able to play both the piano and the organ, making it possible to compare performance in the two groups using the same instrument. Mean age of the improvising group was 46 years (SD: ±14); one subject was left-handed, one subject had perfect pitch. In the score-dependent group, mean age was 39 (SD: ±13); two subjects were left-handed, three had perfect pitch (see Table 1). There was no significant difference between the two groups in age (T-value: 1.15; p = 0.265) or years of professional experience (T-value: 0.96; p = 0.347). Professional experience was defined as the number of years since completion of the propadeutic exam which, in the Dutch educational system, marks formal admission to the second year of the conservatory.

**Table 1:** Subject attributes.

Group	Right-handed	Perfect pitch	Age	Years of professional experience
Improvising	11/12	1 subject	46 (±14)	22 (±13)
Score-dependent	8/10	3 subjects	39 (±13)	17 (±13)

Hand dominance, perfect pitch, age and years of professional experience (expressed in number of years since completion of the propadeutic exam): mean (±SD).

### Experimental procedure

Subjects performed six different tasks on a digital piano on the basis of aural perception of short (±6s) excerpts from polyphonic pieces in the two-part, tonal style consisting of a bass and a treble voice of equal rhythmic and melodic salience. For examples of music excerpts, see: S1 Transcriptions. Excerpts were presented in six blocks, each devoted to a separate task. With the exception of the first excerpt in each block which was used to rehearse the task, the excerpts could all be considered unfamiliar, having been selected from pieces composed specifically for the fMRI experiment [26] and therefore heard only once in the scanner.

Twenty-seven different excerpts were presented. Excerpts were used only once during the experiment with the exception of six excerpts without aural feedback in block 1 and 2 which were later presented (with feedback) in a different tonality, four as the first motif of one of the sequences in block 5 and two for a transposition task. Each excerpt was comprised of a complete motif or phrase.

### Tasks and conditions

Six tasks were performed on a digital piano under one of two conditions: with aural feedback or without (silent keyboard mode). Tasks were presented in six blocks, each containing five to eight excerpts (the number of excerpts is indicated in parentheses):

1. Play along [no aural feedback] (n = 8): subjects were instructed to play together (simultaneously) with two consecutively presented recordings of the excerpt, without aural feedback. The tonality, which was the same for all excerpts, was announced before the task started.
2. Replicate [no aural feedback] (n = 5): subjects were instructed to listen to the excerpt twice and then play it themselves, without aural feedback. The tonality, which was the same for all five examples, was announced before the task started.
3. Replicate and then transpose to the relative minor [aural feedback] (n = 5): subjects were instructed to listen to the excerpt twice, a) play it once in the same (major) key and then b) transpose it to the relative minor.
4. Replicate, adding inner voices [aural feedback] (n = 5): subjects were instructed to listen to the excerpt twice and then play it, adding inner voices belonging to the harmony.
5. Replicate [aural feedback] (n = 7): subjects were instructed to listen to the excerpt twice and then play it in the same key. Subjects were informed that the excerpts used in this task each contained a sequential repetition. For an example, see excerpt 5 (S1 Transcriptions).
6. Replicate and then transpose [aural feedback] (n = 5): subjects were instructed to listen to the excerpt twice, a) play it once in the same key (all excerpts were in g minor) and then b) transpose it to e minor.

While all tasks and conditions involved a form of replication of the aural model, they were also designed to promote recruitment of implicit knowledge of music and music syntax. Performance without aural feedback, for example, was designed to elicit top-down recruitment of procedural knowledge of the tonality. The two-part style used in all tasks would induce disambiguation of the harmony based on prior experience, and the obligation to add inner voices in block 4 might enhance this effect. The sequential repetition found in all excerpts in block 5 was designed to recruit knowledge of both harmony and tonality, just as the transposition tasks in blocks 3 and 6.

### Data acquisition

The music excerpts were performed by one of the researchers (RH), a professional pianist, on an AKAI piano-action MPK88 MIDI (Musical Instrument Digital Interface) controller, without pedal, using the Steinberg ‘The Grand 3’ Yamaha C7, and recorded as midi sequences in Cubase AI5 using a Steinberg CI2 audio interface. Instead of recording the audio signal, MIDI registers key depression and velocity, allowing digital analysis. The choice not to use pedal was motivated by the fact that it might compromise the independence of the voices in the two-part tonal style and/or confound the analysis of the midi sequences. Every effort was made to achieve an ecologically valid concert performance despite the use of an electronic instrument.

Data acquisition made use of the same instrument used to record the aural model. Audio was presented with Stagg SHP-2300 stereo headphones. Subjects familiarized themselves with the keyboard prior to acquisition and adjusted the volume themselves. Before the experiment started, the protocol was explained in detail, block by block, making use of printed instruction material. Subjects then rehearsed the first excerpt from each block. Subjects were instructed that each of the six blocks would consist of at least five examples, one previously rehearsed excerpt and four or more unfamiliar excerpts.

During acquisition, the instructions for each block were repeated before it began, and the subject was reminded that the first example had already been rehearsed. An oral prompt announced that the recording was about to begin. Subjects then heard the pitches from the first beat of the ensuing recording, or if the example began with an upbeat, both the upbeat and the first beat. After a few seconds a woodblock (tuned to the first beat) would indicate the tempo by playing a full bar in the tempo of the recording, one note to a beat. Then the presentation of the music would begin. Subjects were allowed to hear each example twice, with an empty bar between presentations. During the empty bar, the woodblock kept time, playing on every beat. The amount of time given to perform each task was three times the length of the aural model.

### Analysis

Analysis consisted of a comparison between the original midi sequences used as the aural model and the midi sequences produced by the subjects during acquisition. The original midi sequences of the aural model were edited into a separate treble and bass midi sequence in Cubase. In addition, for the transposition tasks, the sequences were transposed to the new key in Cubase and not re-recorded, in order to preserve timing and expression. The midi sequences produced during acquisition were also edited into a separate treble midi sequence and a bass sequence. The ‘finding of the right key’ was edited out of the midi sequence, as well as false starts: subjects playing the first few beats, stopping and then beginning again. In a few cases, subjects did not respond to the aural model and in a few cases, only one voice was played, usually the treble. The inner voices from block 4 were edited out of the midi sequences as well as all other extra improvised voices.

In block 1, a large number of subjects did not play along with the first presentation of each excerpt. To avoid a bias, this first presentation was discarded before analysis, not only for the subjects who did not respond, but also for the subjects who immediately played along with the first presentation. The rehearsed excerpt from each block was also discarded before analysis. Therefore, the total number of midi sequences came to a maximum of thirty-seven per subject, per voice. After editing, the average number of midi sequences was 35.8 (±1.5) for the treble voice and 35.4 (±1.7) for the bass. If no midi sequence was acquired, it was treated as a missing value.

The similarity of the aural model and the performance of the subject was determined by the construction of an alignment. This approach has often been used in musicology, especially in folk song research where it has been used to study the variability of melodies in oral transmission [27]. Algorithmic alignment of melodies was proposed by Mongeau and Sankoff [28]. In this approach, the steps to construct an alignment of two melodies are explicitly formulated such that it can be executed by a computer. The general procedure is to provide the algorithm with two sequences of symbols (notes in our case), after which the algorithm will return the optimal alignment of the two sequences together with a score indicating the extent to which the sequences were able to be aligned. In the present study, we used this score as a proxy for the similarity between the aural model and the subject’s performance, both of which are represented as sequences of MIDI events.

In recent years, alignment algorithms have often been employed in Computational Musicology and Music Information Retrieval [29–31]. The aim of an alignment algorithm is to find the (or one of the) alignments(s) with the highest score. Since the solution space is quite large, a dynamic programming approach is generally taken to find the optimal alignment efficiently. In the simplest form, the optimal alignment and its score are found by filling a matrix D recursively according to:

$$D(i,j)=\text{max}\{\begin{array}{l} D(i-1,j-1)+S(x_{i},y_{j})\\ D(i-1,j)+\gamma \\ D(i,j-1)+\gamma \end{array},i\in \left[1,\ldots n\right],j\in \left[1,\ldots m\right]$$

in which x: *x*_1_,…,*x*_*i*_,…,*x*_*n*_, and y: *y*_1_,…,*y*_*j*_,…,*y*_*m*_ are the sequences to be aligned, *S*(*x*_*i*_, *y*_*j*_) is a similarity measure for arbitrary symbols, and γ is the (fixed) gap score (the gap score is the numerical score awarded to a note in the replication of the aural model that does or does not correspond to a note in the aural model). *D*(0,0) = 0, *D*(*i*,0) = *iγ* and *D*(0,*j*) = *jγ*. *D*(*i*,*j*) contains the score of the optimal alignment up to symbols *x*_*i*_ and *y*_*j*_ of sequence x and y respectively and therefore *D*(*n*,*m*) contains the score of the optimal alignment of the complete sequences. The algorithm has both time and space complexity *O*(*nm*), which is quadratic. This algorithm is known as the Needleman-Wunsch algorithm [32]. For further details, see: S1 Appendix.

To apply this algorithm to melodies, or in this case midi sequences, the abstract elements of the algorithm that need to be defined are 1) the symbols, 2) the substitution score function, and 3) the gap score γ. In the present study, as we were dealing with MIDI, we took each element from the midi sequence (onset, pitch, duration) as a symbol. We subsequently determined a substitution score function *S*(*x*_*i*_,*y*_*j*_) and the gap score γ. The intuitive meaning of the substitution score function is: the higher the substitution score of two symbols, the more we want them to be aligned. In general, this implies that the substitution score function will be defined as a similarity measure for symbols. To define the function, we can of course use different properties of the notes. For the present study, we used exact pitch and IOR (interonset interval ratio). Both are available in, or computable from, the MIDI-input. To represent pitch, we used the MIDI-representation, which basically corresponds to the indices of the keys of the keyboard in which a^1^ (A440) = 69.

The IOR of a given note is the ratio between the IOI (interonset interval) of the note and the IOI of the previous note, where the IOI of a note is defined as the difference in time between the onset of one note and the onset of the next. The IOR can be considered to be the relative duration of a given note with respect to the previous note. For the last note in the sequence we defined the IOI as the duration of that note. For the first note in the sequence, we set IOR to 1.

We defined two substitution score functions:

$$S_{pitch}\;(x_{i},y_{j})=\{\begin{array}{l} \;1\text{ if }p(x_{i})=p(y_{j})\\ -1\text{ if }p(x_{i})\neq p(y_{j}) \end{array}$$

in which *p*(*s*) is the pitch of symbol x in MIDI encoding, and

$$S_{ior}(x_{i},y_{j})=\{\begin{array}{l} -1+2*(ior(y_{j})/ior(x_{i}))\text{ if }ior(x_{i})\;\geq \;ior(y_{j})\\ -1+2*(ior(x_{i})/ior(y_{j}))\text{ if }ior(x_{i})\;<\;ior(y_{j}) \end{array}$$

where *ior*(*x*_*i*_) = *ioi*(*x*_*i*_)/*ioi*(*x*_*i*-1_), in which *ioi*(*x*_*i*_) is the time difference between the onsets of *x*_*i*_ and *x*_*i* + 1_. We defined the gap score as γ = -0.5 for exact pitch and γ = 0 for IOR. In the event we used *S*_*pitch*_ we obtained a value for the similarity of the aural model and the recorded midi sequence with respect to the sequence of pitches, and when we used *S*_*ior*_ we got a value for the similarity of the aural model with the recorded midi sequence with respect to the sequence of IORs, which reflects rhythmic similarity.

Since the score of an alignment depends on the length of the midi sequences, normalization is needed to compare different alignment scores. Otherwise, the alignment of two similar long sequences would result in a much higher score than the alignment of two short sequences. We therefore divided the alignment score by the length of the alignment, which is the length of sequence x increased with the number of gaps inserted in x (or the length of sequence y increased with the number of gaps inserted in y). Thus, an exact match resulted in a score of 1, as the maximum value of our substitution score functions is 1, and no gaps are needed, causing the score of the alignment to equal the length of the sequences. Anything less than an exact match resulted in a score lower than 1. The scores that are reported in this paper are the normalized alignment scores.

Our main goal was assessment of the differing ability of improvising and score-dependent musicians to replicate aurally perceived music at the piano. Accordingly, mean alignment scores for the four variables (treble exact pitch, treble IOR (interonset ratio), bass exact pitch, and bass IOR) were subjected to a one-way multivariate analysis of variance (MANOVA) to determine significance of the difference between groups. Subsequently, differences of means were tested for each of the four variables using one-way ANOVA. Interactions between the factors group (improvising, score-dependent), voice (treble, bass), and parameter (exact pitch, IOR) were studied using a three-way mixed (between-subjects/within-subjects/within-subjects) analysis of variance (ANOVA).

For the comparison of replication and transposition, the replication tasks from block 3 and 6 (3a and 6a) were contrasted with the transposition tasks from the same blocks (3b and 6b), based on identical stimuli. Two-way mixed ANOVA was used to investigate the interaction between group (between-subjects) and task (within-subjects) for each of the four variables. Similarly, for the comparison of performance with and without aural feedback, the replication tasks from block 1 and 2 without feedback were contrasted with all replication tasks from blocks 3–6 with aural feedback (3a, 4, 5, and 6a). Again, two-way mixed ANOVA was used to investigate the interaction between group (between-subjects) and condition (within-subjects) for each of the four variables.

## Results

Summarizing tasks and conditions, improvising and score-dependent subjects listened to short music excerpts composed in the two-part tonal style and performed various replication tasks, either 1) playing along with the excerpt, 2) listening and then replicating it in the same key, 3) listening and replicating a major-key excerpt, first in the same key and then in the relative minor, 4) listening and replicating the excerpt while adding inner voices, 5) listening to an excerpt containing a sequential repetition and replicating it in the same key, or 6) listening and replicating the excerpt, first in the same key and then in a different key. Tasks were performed under two contrasting conditions: with aural feedback (blocks 3–6) or without (blocks 1 and 2). Alignment scores were computed for exact pitch and IOR (which reflects rhythmic similarity) for the treble and bass voices separately. Mean alignment scores of the participants are presented in the **Supporting Information** (S1 Alignment Scores). MIDI sequences of the aural model and the individual participants have been made available in the **Supporting Information** (S1 Research Data).

### Group: improvising vs. score-dependent musicians

**Fig 1:**
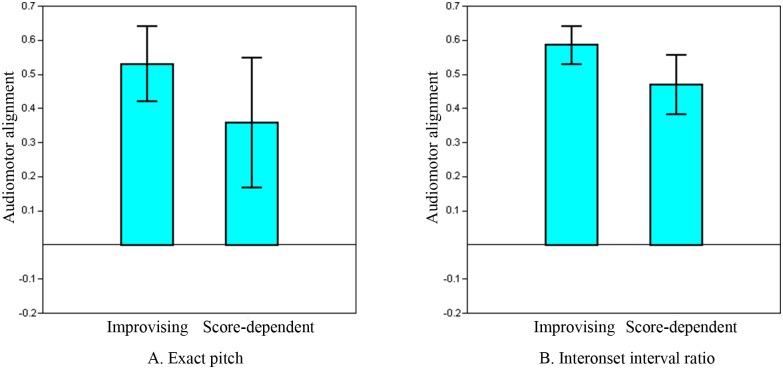
Treble audiomotor alignment: comparison of groups. Improvising vs. score-dependent musicians (mean ± SD). The comparison concerns the treble voice, all tasks (both conditions). A: exact pitch: improvising > score-dependent and B: IOR (interonset interval ratio): improvising > score-dependent. See Table 2 for exact values and parameters of significance tests.

One-way multivariate analysis of variance (MANOVA) revealed a significant difference between improvising and score-dependent musicians based on their combined audiomotor alignment scores, *F*(4, 17) = 3.309, p = 0.035. Subsequent one-way ANOVA indicated that improvising musicians’ mean treble alignment scores were significantly higher than those of score-dependent musicians, both for exact pitch and IOR (Fig 1, see Table 2 for exact values and parameters of significance tests). Mean bass alignment scores were also significantly higher for improvising musicians, however only for IOR. The range of alignment scores was larger for score-dependent musicians, particularly for exact pitch, although there was no significant difference of variance. Score-dependent musicians exhibited not only the lowest scores, but also a few of the highest, both in the treble and the bass.

**Table 2:** Audiomotor alignment: comparison of groups.

Parameter	Voice	Improvising	Score-dependent	F	p-value
Exact pitch	Treble	0.5311 (±0.11)	0.3592 (±0.19)	7.028	0.015
IOR	Treble	0.5864 (±0.06)	0.4699 (±0.09)	14.534	0.001
Exact pitch	Bass	0.1323 (±0.19)	0.0191 (±0.21)	1.734	0.203
IOR	Bass	0.4560 (±0.05)	0.3576 (±0.10)	8.535	0.008

Audiomotor alignment: improvising > score-dependent musicians. Values represent the group mean (± SD). Alignment is expressed in an audiomotor alignment score, maximum = 1 (see methods). IOR: interonset interval ratio. Significance was determined using one-way ANOVA.

### Interactions

**Fig 2:**
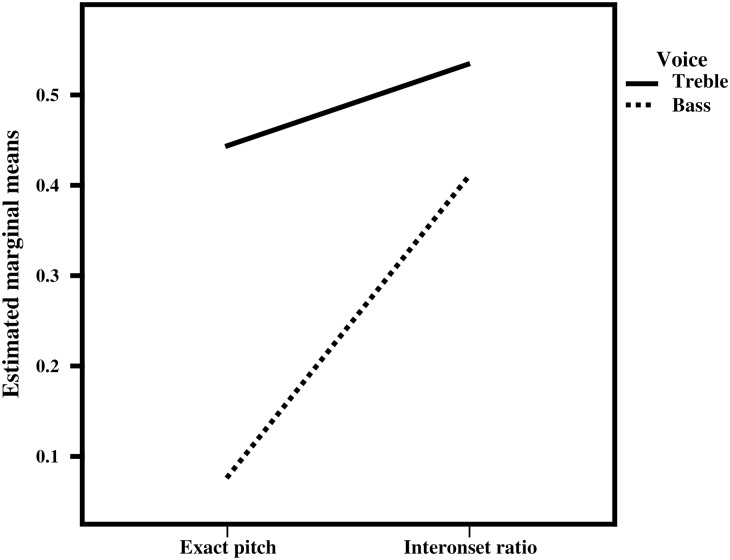
Interaction between parameter and voice. Alignment scores were higher for the treble voice (top line) than for the bass (bottom line), both for exact pitch and IOR. The effect of parameter on alignment was larger in the bass (steeper bottom line) than in the treble voice. Significance of the interaction, *F*(1, 20) = 102.636, p < 0.0001, was determined using three-way mixed ANOVA.

A three-way mixed (between-subjects, within-subjects, within-subjects) ANOVA was conducted to investigate interaction between group, voice, and parameter which, however, was not observed. A significant two-way interaction was observed between parameter and voice, *F*(1, 20) = 102.636, p < 0.0001 (see Fig 2).

In addition to the two-way interaction, a statistically significant main effect of both voice (treble > bass), *F*(1,20) = 116.508, p < 0.0001 and parameter (IOR > exact pitch), *F*(1,20) = 66.584, p < 0.0001 was observed. The latter was not further explored as we considered a pitch-rhythm comparison to be conceptually non-informative. One-way ANOVA indicated that the effect of voice (treble > bass) pertained to both exact pitch and IOR and was significant for both groups (Table 3).

**Table 3:** Treble vs. bass.

Group	Parameter	Treble	Bass	F	p-value
All subjects	Exact pitch	0.4530 (±0.17)	0.0808 (±0.20)	42.809	< 0.0001
All subjects	IOR	0.5335 (±0.09)	0.4113 (±0.09)	19.556	< 0.0001
Improvising	Exact pitch	0.5311 (±0.11)	0.1323 (±0.19)	39.419	< 0.0001
Improvising	IOR	0.5864 (±0.06)	0.4560 (±0.05)	37.841	< 0.0001
Score-dependent	Exact pitch	0.3592 (±0.19)	0.0191 (±0.21)	14.241	0.001
Score-dependent	IOR	0.4699 (±0.09)	0.3576 (±0.10)	6.822	0.018

Audiomotor alignment: treble > bass, all tasks and conditions. Values represent the group means (±SD). Alignment is expressed in an audiomotor alignment score, maximum = 1 (see methods). IOR: interonset interval ratio. Significance was determined using one-way ANOVA.

### Task: replication vs. transposition

**Fig 3:**
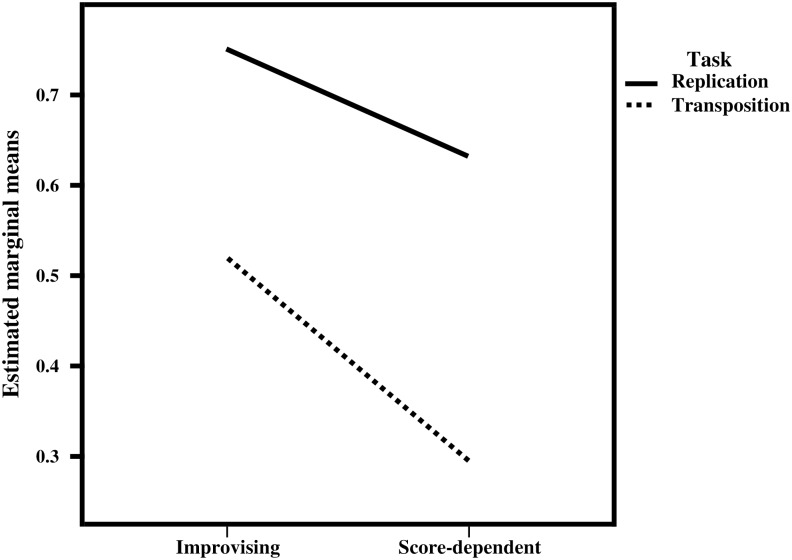
Interaction between group and task: treble exact pitch. Treble exact pitch alignment scores were higher for replication (top line) than for transposition (bottom line). The effect of group (improvising > score-dependent) was larger for transposition (steeper bottom line) than for replication in the original key. Significance of the interaction, *F* (1, 20) = 4.483, p = 0.047, was determined using two-way mixed ANOVA.

In blocks 3 and 6, the same stimulus was used for two different tasks, enabling a direct comparison between replication (in the original key) and transposition (to a different key or to the relative minor). Two-way mixed ANOVA revealed significant interaction between group (between-subjects) and task (within-subjects), but only for treble exact pitch, *F* (1, 20) = 4.483, p = 0.047 (Fig 3). A significant main effect was found for task (treble exact pitch: replication > transposition), *F* (1, 20) = 121.364, p < 0.00001. As can be seen in Fig 3, the difference in replication of exact pitch in the original key by the two groups of musicians was relatively small (top line). The steeper bottom line, however, illustrates the fact that score-dependent musicians performed less well when transposing to a different key. Perusal of individual treble alignment scores revealed that only two of the twelve improvising musicians actually exhibited significantly lower alignment scores for treble exact pitch transposition, compared to replication, while six out of ten score-dependent musicians exhibited significantly lower scores for transposition versus replication. With the exception of bass exact pitch, alignment scores for transposition were all significantly higher in improvising musicians, similar to the group difference for the replication of treble and bass IOR (Table 4).

**Table 4:** Improvising vs. score-dependent: task.

Task	Voice	Parameter	Improvising	Score-dependent	F	p-value
Replication	Treble	Exact pitch	0.7497 (±0.08)	0.6333 (±0.18)	4.115	0.056
Transposition	Treble	Exact pitch	0.5204 (±0.14)	0.2948 (±0.26)	6.654	0.018
Replication	Treble	IOR	0.6433 (±0.04)	0.5345 (±0.08)	18.930	< 0.0001
Transposition	Treble	IOR	0.6260 (±0.08)	0.4849 (±0.14)	9.292	0.006
Replication	Bass	Exact pitch	0.2259 (±0.22)	0.1231 (±0.31)	0.827	0.374
Transposition	Bass	Exact pitch	0.0884 (±0.26)	-0.0174 (±0.30)	0.788	0.385
Replication	Bass	IOR	0.4891(±0.07)	0.3967 (±0.13)	4.702	0.042
Transposition	Bass	IOR	0.4780 (±0.08)	0.3662 (±0.14)	5.713	0.027

Group comparison per task: improvising > score-dependent. Values represent the group mean (± SD). Alignment is expressed in an audiomotor alignment score, maximum = 1 (see methods). IOR: interonset interval ratio. Means were compared using one-way ANOVA.

### Condition: aural feedback vs. no aural feedback

**Fig 4:**
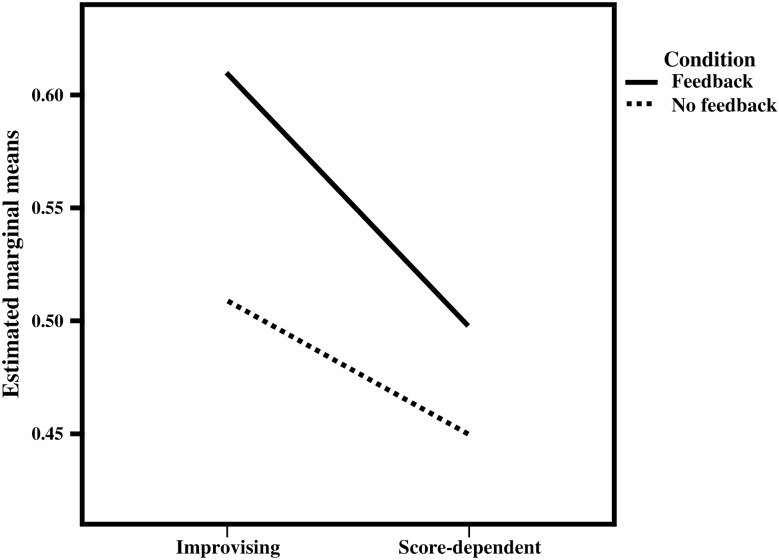
Interaction between group and condition: treble IOR. Treble IOR scores were higher for performance with feedback (top line) than without feedback (bottom line). The effect of group (improvising > score-dependent) was larger for performance with feedback (steeper top line) than for performance without feedback. Significance of the interaction, *F*(1, 20) = 6.254, p = 0.021, was determined using two-way mixed ANOVA.

In blocks 1 and 2, subjects had no access to aural feedback during performance of the tasks. Two-way mixed ANOVA revealed interaction between group (between-subjects) and condition (within-subjects), but only for treble IOR, *F*(1, 20) = 6.254, p = 0.021 (Fig 4). Subsequently, one-way ANOVA indicated that improvising musicians scored higher than score-dependent musicians on treble exact pitch and IOR as well as bass IOR, both with and without feedback (Table 5).

**Table 5:** Improvising vs. score-dependent: condition.

Condition	Voice	Parameter	Improvising	Score-dependent	F	p-value
Feedback	Treble	Exact pitch	0.6263 (±0.10)	0.4717 (±0.17)	7.099	0.015
No feedback	Treble	Exact pitch	0.4972 (±0.16)	0.3209 (±0.21)	4.934	0.038
Feedback	Treble	IOR	0.6219 (±0.05)	0.4961 (±0.08)	20.195	< 0.0001
No feedback	Treble	IOR	0.5217 (±0.07)	0.4480 (±0.08)	5.208	0.034
Feedback	Bass	Exact pitch	0.1682 (±0.20)	0.0589 (±0.24)	1.336	0.261
No feedback	Bass	Exact pitch	0.1635 (±0.19)	0.0424 (±0.17)	2.494	0.130
Feedback	Bass	IOR	0.4715 (±0.06)	0.3698 (±0.11)	8.354	0.009
No feedback	Bass	IOR	0.4378 (±0.0.04)	0.3490 (±0.10)	8.466	0.009

Group comparison per condition: improvising > score-dependent (treble alignment). Values represent the group mean (± SD). Alignment is expressed in an audiomotor alignment score, maximum = 1 (see methods). IOR: interonset interval ratio. Means were compared using one-way ANOVA.

### Correlations

No significant correlations were found between mean alignment scores and either age (treble exact pitch: r_s_ = 0.02; treble IOR: r_s_ = 0.19; bass exact pitch: r_s_ = -0.22; bass IOR: r_s_ = -0.08) or years of professional experience, expressed in number of years since completion of the propadeutic exam (treble exact pitch: r_s_ = 0.02; treble IOR: r_s_ = 0.17; bass exact pitch: r_s_ = -0.14; bass IOR: r_s_ = -0.02). One of the highest scoring organists was actually still completing his bachelor in organ performance at the time of the study.

## Discussion

The results of the present study indicate that improvising musicians can be distinguished from their score-dependent counterparts on the basis of their superior ability to replicate both the pitch and the rhythm of aurally perceived music on their instrument. While this ability is particularly evident in the treble voice, it also extends to the bass voice in the temporal domain. Higher treble alignment scores in improvising musicians could be associated with their superior ability to replicate both the pitch and rhythm of the treble voice in other tonalities (aural transposition). With the exception of treble IOR, aural feedback did not contribute significantly to higher alignment scores in improvising musicians, however, a possible effect of aural feedback on transposition was not assessed. The higher audiomotor alignment scores found here can be seen as evidence of enhanced audiomotor transformations, a notion that is supported by the significantly larger activation of the right dorsal parietal-premotor network identified with fMRI in improvising musicians while the participants imagined playing along with a recording or covertly assessed the quality of the performance [26].

The superior ability to replicate aurally perceived music ‘by ear’, particularly the ability to do so in different contexts, for example during aural transposition, may possibly be associated with enhanced employment of procedural knowledge of music syntax during performance. SRT (Serial Reaction Time) studies have demonstrated that implicit knowledge of low-level action syntax can be acquired non-consciously during the mere repetition of motor sequences [33–35]. SRT studies of hierarchically more complex syntax, however, show that mere repetition is not sufficient for implicit acquisition to take place [36–38].

The apparent distinction between low- and high-level syntax is reflected by the existence of specific processing networks in the brain dedicated to low- and high-level syntax. Imaging studies indicate that processing of low-level syntax activates a ventral network comprised of the frontal operculum and anterior temporal cortex [39] while complex syntax additionally activates a dorsal network involving posterior inferior frontal gyrus (caudal Broca) and posterior superior temporal cortex [40].

‘String parsing’ has been proposed as the mechanism by which ‘program-level imitation’ of behavior leads to the acquisition of hierarchically complex syntax [41]. It also offers a ‘parsimonious’ explanation for the beneficial effects of improvisation on the acquisition of hierarchically complex music syntax. An important characteristic of the practice methods employed in classical music is the frequent repetition of the notes in the exact order in which they are to be played. While repetition may be expected to lead primarily to segmentation and chunking of the sequence i.e. to the implicit acquisition of low-level syntax, syntax-congruent manipulation of the serial order while playing ‘by ear’ might lead to implicit parsing of the hierarchical structure.

The large individual differences in alignment scores found in the score-dependent group suggest that practice strategies in classical music might not be as uniform as one would think. It would seem that practice methods fostering implicit, non-conscious audiomotor learning are actually employed by a minority of score-dependent musicians and can be said to have a beneficial effect on the implicit acquisition of hierarchical music syntax. Although improvisation, like immersion in language acquisition [42], is a fertile ground for the type of implicit, non-conscious learning that is involved in audiomotor integration [43], parsing of the hierarchical structure is apparently also achieved during the practice of repertoire, given the right approach.

Treble alignment scores for replication were significantly higher than for transposition in both groups, but only for exact pitch, suggesting that transposition may not be just another form of pitch replication. Recent studies have implicated the right intraparietal sulcus (IPS) in transposition [21], retrograde musical transformations [22], and pitch-to-space transformations [23]. The fact that, during transposition, improvising musicians scored significantly higher than score-dependent musicians on both treble exact pitch and IOR indicates that they are more capable of performing such pitch-to-space transformations. It seems quite likely that improvising musicians’ greater success in replicating aurally perceived music at the original pitch is also based on the same type of audiomotor transformations they are employing during transposition. The smaller difference between replication and transposition exhibited by most improvising musicians in this study supports that view.

The observation that treble alignment scores were significantly higher than bass alignment scores, despite the use of two-part polyphonic excerpts, corroborates the high-voice superiority effect found in both behavioral and neural studies. A higher-pitch advantage for melody recognition was found in infants [44] as well as in musically trained and untrained individuals [45]. Auditory brainstem response to intervals has revealed heightened responses to harmonics of the upper tone [46]. MMN response to higher-pitched deviants is larger and earlier [47–48]. Seventh-month old infants show earlier and larger MMN to changes in the higher voice [49]. MMN in even younger (3-month old) infants was smaller and later than 7 month infants, but size of MMN difference was similar across ages, supporting the hypothesis of an innate origin of the high-voice superiority effect [50].

The high-voice superiority effect has been shown to be subject to neuroplasticity. MMN caused by pitch deviants in the bass has been found to be equal (but not larger) to that elicited by the treble voice in double bass players [51]. In addition, lower-voice superiority has been found for temporal deviants in players of bass instruments [52]. The significantly higher scores for bass IOR observed in improvising musicians and the fact that bass IOR alignment scores were higher than those of score-dependent musicians both with and without aural feedback, suggests that they may also be subject to a lower-voice superiority effect. This is one group difference that could possibly be attributed to the instrument the subjects played, rather than to the practice of improvisation. Organists commonly use the pedals to play the bass line, while pianists incorporate the bass line in the left-hand part. In that sense, organists can be said to play a bass instrument and may therefore also be subject to the lower-voice superiority effect.

An advantage of aural feedback was observed for the replication of treble IOR, but only for improvising musicians. At first sight, this might seem to conflict with studies that have demonstrated that musicians are largely independent of aural feedback [53]. Performance without aural feedback is not only as accurate, but also almost as expressive as with feedback [54]. The concept that aural feedback might not be essential is supported by a study using event-related potentials (ERP) during the performance of memorized music, revealing early error signaling, before the actual error, independent of aural feedback [55]. Aural feedback has been shown to be more important during the learning phase than during music performance itself [56].

While musicians are quite able to perform without aural feedback, asynchronously altered feedback (AAF) may compromise performance during both singing and playing. Delaying feedback until the next tone is being sung or played (serial shift), however, compromises performance in singers but not in score-dependent pianists [57]. While the singers in the cited study had learned the melodies aurally, the pianists, being unable to play the melodies ‘by ear’, had learned them from music notation. The authors argue that the disruptive effects of altered feedback are ‘based on abstract, effector-independent, associations between perception and action’, suggesting that action-perception associations are stronger in singers than in score-dependent pianists. Though the experimental paradigm was considerably different, stronger action-perception associations in improvising musicians might also be responsible for the larger benefit from aural feedback experienced by improvising musicians in the present study during replication of the rhythm of the melody. Further study is necessary to determine the effect of feedback on aural transposition.

## Conclusions

The present study has found behavioral evidence for superior audiomotor transformation during the replication and particularly the transposition of aurally perceived music in improvising musicians. These results are consistent with the associated fMRI study [26], providing arguments suggesting that improvisation supports audiomotor learning in music performance. The present findings underscore the notion that the gradual disappearance of improvisational task requirements in the field of classical music since the middle of the nineteenth century [58] has had a large impact not only on concert practice but, more importantly, also on the audiomotor characteristics of the musicians themselves. Nevertheless, the high alignment scores exhibited by a small number of score-dependent musicians indicate that, besides improvisation, specific practice strategies may also have an important impact on audiomotor integration [59].

## Supporting Information

S1 Alignment Scores (ZIP)

S1 Appendix Alignment. (PDF)

S1 Research Data (ZIP)

S1 Transcriptions Music excerpts. (PDF)
