# Supplementary material for: Behavioral Quantification of Audiomotor Transformations in Improvising and Score-Dependent Musicians
Source: PLoS One. 2016 Nov 11;11(11):e0166033. doi: 10.1371/journal.pone.0166033 (PMC5105996; doi:10.1371/journal.pone.0166033)
Supplement: S1 Alignment Scores — (ZIP) [file pone.0166033.s001.zip › Alignment_scores_10.pdf]

Alignment scores 10. IOR bass alignment: feedback/no feedback.

GROUP	SUBJECT	VOICE	CONDITION	BLOCKS	Min	Max	Mean	Stand. dev	Median	25 prcntil	75 prcntil
Improvising	N3851	bass	feedback	3a, 4, 5, 6a	0.263001	0.799	0.5062308	0.163947	0.502913	0.3564485	0.640952
Improvising	N3933	bass	feedback	3a, 4, 5, 6a	0.361234	0.8916	0.5615973	0.1554216	0.550727	0.4489728	0.680338
Improvising	N3938	bass	feedback	3a, 4, 5, 6a	0.178055	0.90139	0.4628311	0.2000689	0.4370435	0.321443	0.5944325
Improvising	N3974	bass	feedback	3a, 4, 5, 6a	0.205268	0.547756	0.3780441	0.1158592	0.3699835	0.2808275	0.4851468
Improvising	N4223	bass	feedback	3a, 4, 5, 6a	0.316048	0.66799	0.4608291	0.1336067	0.4396575	0.326898	0.5859885
Improvising	N4229	bass	feedback	3a, 4, 5, 6a	0.240895	0.934686	0.5417631	0.2260775	0.4920995	0.362759	0.8088112
Improvising	N4258	bass	feedback	3a, 4, 5, 6a	0.205095	0.712617	0.3948263	0.1280099	0.386614	0.2871585	0.4799605
Improvising	N4486	bass	feedback	3a, 4, 5, 6a	0.226248	0.725544	0.4174884	0.1304376	0.404811	0.3250415	0.4785695
Improvising	N4549	bass	feedback	3a, 4, 5, 6a	0.172533	0.85658	0.4678096	0.1454418	0.4779175	0.405203	0.5091925
Improvising	N4774	bass	feedback	3a, 4, 5, 6a	0.245046	0.809039	0.4640428	0.161501	0.4291365	0.3623445	0.5238027
Improvising	N4869	bass	feedback	3a, 4, 5, 6a	0.2528	0.848215	0.4772202	0.188682	0.398796	0.3495497	0.6286832
Improvising	N5692	bass	feedback	3a, 4, 5, 6a	0.296407	0.82906	0.5247368	0.1877434	0.486964	0.360351	0.7001245
Score-dep.	N4429	bass	feedback	3a, 4, 5, 6a	0.251381	0.787376	0.398599	0.1371464	0.363536	0.2885258	0.456549
Score-dep.	N4517	bass	feedback	3a, 4, 5, 6a	0.194138	0.684141	0.3701342	0.1641394	0.3121455	0.238272	0.464383
Score-dep.	N4588	bass	feedback	3a, 4, 5, 6a	0.24381	0.538372	0.3442322	0.0933142	0.3211935	0.260696	0.3874
Score-dep.	N4615	bass	feedback	3a, 4, 5, 6a	0.253742	0.698988	0.4855392	0.1391089	0.5348055	0.37504	0.57586
Score-dep.	N4657	bass	feedback	3a, 4, 5, 6a	0.111111	0.43927	0.2444325	0.0947934	0.244984	0.165848	0.3089005
Score-dep.	N5064	bass	feedback	3a, 4, 5, 6a	0.20806	0.67231	0.4101579	0.1392126	0.40842	0.2983235	0.5313288
Score-dep.	N5480	bass	feedback	3a, 4, 5, 6a	0.307024	0.868265	0.5477222	0.1878476	0.508559	0.3860438	0.7317255
Score-dep.	N5484	bass	feedback	3a, 4, 5, 6a	0.0555556	0.39583	0.1827548	0.0974588	0.198174	0.1010101	0.249808
Score-dep.	N5783	bass	feedback	3a, 4, 5, 6a	0.0978776	0.578298	0.3783424	0.1295708	0.3964325	0.2852938	0.463634
Score-dep.	N6128	bass	feedback	3a, 4, 5, 6a	0.148362	0.581707	0.3357628	0.1211254	0.329494	0.2655332	0.3922558

Alignment scores 10. IOR bass alignment: feedback/no feedback.

GROUP	SUBJECT	VOICE	CONDITION	BLOCKS	Min	Max	Mean	Stand. dev	Median	25 prcntil	75 prcntil
Improvising	N3851	bass	no feedback	1,2	0.18809	0.708579	0.4596925	0.1672576	0.439373	0.325277	0.608348
Improvising	N3933	bass	no feedback	1,2	0.276684	0.813205	0.4766466	0.1541957	0.45601	0.373597	0.581082
Improvising	N3938	bass	no feedback	1,2	0.193388	0.874592	0.4714269	0.2199064	0.434396	0.314561	0.570817
Improvising	N3974	bass	no feedback	1,2	0.125	0.832737	0.435251	0.2176155	0.4366655	0.240978	0.5650593
Improvising	N4223	bass	no feedback	1,2	0.214543	0.93873	0.4983614	0.2032971	0.506609	0.380139	0.589175
Improvising	N4229	bass	no feedback	1,2	0.177575	0.923225	0.4088747	0.1980553	0.359467	0.301847	0.418315
Improvising	N4258	bass	no feedback	1,2	0.111111	0.587089	0.3660906	0.1511453	0.385497	0.290237	0.459266
Improvising	N4486	bass	no feedback	1,2	0.111111	0.532858	0.3823443	0.1287695	0.427551	0.304221	0.461237
Improvising	N4549	bass	no feedback	1,2	0.166667	0.629203	0.4248925	0.1278608	0.456142	0.366282	0.493524
Improvising	N4774	bass	no feedback	1,2	0.221975	0.790456	0.4680663	0.1555167	0.46018	0.345469	0.556476
Improvising	N4869	bass	no feedback	1,2	0.197511	0.783723	0.4377551	0.1968312	0.414661	0.270559	0.530554
Improvising	N5692	bass	no feedback	1,2	0.1	0.703719	0.424445	0.1553234	0.451078	0.345321	0.499857
Score-dep.	N4429	bass	no feedback	1,2	0.1	0.754284	0.3805722	0.2046304	0.37666	0.198433	0.495457
Score-dep.	N4517	bass	no feedback	1,2	0.156666	0.658421	0.3263374	0.1472334	0.302557	0.188	0.409559
Score-dep.	N4588	bass	no feedback	1,2	0.216738	0.500911	0.3243158	0.1040132	0.2818625	0.2356585	0.4166655
Score-dep.	N4615	bass	no feedback	1,2	0.111111	0.878335	0.4749377	0.2222619	0.461288	0.337859	0.65386
Score-dep.	N4657	bass	no feedback	1,2	0.1	0.413559	0.2287702	0.1130534	0.196321	0.111111	0.318695
Score-dep.	N5064	bass	no feedback	1,2	0.260504	0.734813	0.4069873	0.1468611	0.360715	0.300193	0.417881
Score-dep.	N5480	bass	no feedback	1,2	0.152611	0.844547	0.4723208	0.1969794	0.466319	0.369317	0.537583
Score-dep.	N5484	bass	no feedback	1,2	0.1	0.33301	0.1658711	0.078213	0.125	0.111111	0.2223755
Score-dep.	N5783	bass	no feedback	1,2	0.111111	0.539445	0.355333	0.1413808	0.392206	0.2612217	0.4562667
Score-dep.	N6128	bass	no feedback	1,2	0.186769	0.54511	0.354291	0.1046305	0.349237	0.261196	0.447963
