# Supplementary material for: Behavioral Quantification of Audiomotor Transformations in Improvising and Score-Dependent Musicians
Source: PLoS One. 2016 Nov 11;11(11):e0166033. doi: 10.1371/journal.pone.0166033 (PMC5105996; doi:10.1371/journal.pone.0166033)
Supplement: S1 Alignment Scores — (ZIP) [file pone.0166033.s001.zip › Alignment_scores_1.pdf]

Alignment scores 1. Exact pitch alignment: treble/bass.

GROUP	SUBJECT	VOICE	BLOCK	Min	Max	Mean	Stand. dev	Median	25 prcntil	75 prcntil
Improvising	N3851	treble	1-6	-0.115385	1	0.6577286	0.3199848	0.7083335	0.5	1
Improvising	N3933	treble	1-6	-0.147059	1	0.6589153	0.3151462	0.769231	0.5	0.875
Improvising	N3938	treble	1-6	-0.214286	1	0.4755695	0.3672924	0.461538	0.190909	0.7916665
Improvising	N3974	treble	1-6	-0.285714	1	0.4795208	0.4139138	0.580128	0.127841	0.8458332
Improvising	N4223	treble	1-6	-0.357143	1	0.5094373	0.3784943	0.5	0.1825395	0.8541665
Improvising	N4229	treble	1-6	0	1	0.6076085	0.3174251	0.576923	0.4251335	0.9423075
Improvising	N4258	treble	1-6	-0.35	1	0.5038191	0.4225431	0.595238	0.1787925	0.875
Improvising	N4486	treble	1-6	-0.263158	1	0.3354021	0.3617346	0.333333	0.0555556	0.625
Improvising	N4549	treble	1-6	-0.25	1	0.3668704	0.35468	0.416667	0.110324	0.5802085
Improvising	N4774	treble	1-6	0	1	0.6328805	0.3102628	0.653846	0.4017855	1
Improvising	N4869	treble	1-6	-0.416667	1	0.5128348	0.4008316	0.571429	0.1770835	0.9375
Improvising	N5692	treble	1-6	-0.1875	1	0.6320751	0.3250176	0.777778	0.3645835	0.869318
Score-dep.	N4429	treble	1-6	-0.416667	1	0.2128424	0.3959569	0.222222	-0.133741	0.527864
Score-dep.	N4517	treble	1-6	-0.166667	1	0.3001828	0.32895	0.230769	0.0491072	0.5
Score-dep.	N4588	treble	1-6	-0.222222	1	0.383625	0.3783851	0.333333	0	0.75
Score-dep.	N4615	treble	1-6	-0.15625	1	0.6662544	0.3483881	0.777778	0.34375	1
Score-dep.	N4657	treble	1-6	-0.392857	0.5	-0.00461	0.2424513	0.0334822	-0.22601	0.1488463
Score-dep.	N5064	treble	1-6	-0.086957	1	0.3639587	0.3065905	0.363636	0.0767046	0.599359
Score-dep.	N5480	treble	1-6	-0.1875	1	0.5722047	0.349338	0.5875	0.3177083	0.8793703
Score-dep.	N5484	treble	1-6	-0.416667	1	0.5050276	0.4189518	0.678571	0.0714286	0.85
Score-dep.	N5783	treble	1-6	-0.2	1	0.3073213	0.3537573	0.28125	0.0290033	0.5
Score-dep.	N6128	treble	1-6	-0.263158	1	0.2855874	0.3324205	0.208333	0	0.5

Alignment scores 1. Exact pitch alignment: treble/bass.

GROUP	SUBJECT	VOICE	BLOCK	Min	Max	Mean	Stand. dev	Median	25 prcntil	75 prcntil
Improvising	N3851	bass	1-6	-0.4375	1	0.2037458	0.3717046	0.1583335	-0.058239	0.4886362
Improvising	N3933	bass	1-6	-0.3	1	0.2422835	0.3885161	0.214286	-0.088889	0.4
Improvising	N3938	bass	1-6	-0.642857	1	0.0437949	0.4193989	-0.035714	-0.281746	0.2261905
Improvising	N3974	bass	1-6	-0.642857	1	0.0584243	0.4114037	-0.045833	-0.168403	0.1916667
Improvising	N4223	bass	1-6	-0.571429	1	0.1167441	0.368104	0.0454545	-0.145834	0.428571
Improvising	N4229	bass	1-6	-0.3125	1	0.4603919	0.4074047	0.444444	0.1525735	0.8484845
Improvising	N4258	bass	1-6	-0.375	0.7	-0.077201	0.2288681	-0.125	-0.25	0
Improvising	N4486	bass	1-6	-0.333333	1	-0.032794	0.2605051	-0.1	-0.208333	0
Improvising	N4549	bass	1-6	-0.444444	0.625	-0.061448	0.2630455	-0.111111	-0.286364	0.0611112
Improvising	N4774	bass	1-6	-0.3	1	0.1620967	0.3599788	0.08	-0.15625	0.3958335
Improvising	N4869	bass	1-6	-0.714286	1	-0.021321	0.3445115	-0.055556	-0.2	0.113248
Improvising	N5692	bass	1-6	-0.357143	1	0.492398	0.4469398	0.5625	0.0416667	1
Score-dep.	N4429	bass	1-6	-0.642857	1	-0.113283	0.3258572	-0.1875	-0.308608	0
Score-dep.	N4517	bass	1-6	-0.4	0.714286	0.0467712	0.2940231	-0.0625	-0.184659	0.1875
Score-dep.	N4588	bass	1-6	-0.5	0.785714	-0.095158	0.2447673	-0.166667	-0.25	0.0595239
Score-dep.	N4615	bass	1-6	-0.333333	1	0.2268909	0.3401409	0.125	-0.050505	0.422619
Score-dep.	N4657	bass	1-6	-0.5625	0.625	-0.144222	0.2800629	-0.208333	-0.322917	-0.1125
Score-dep.	N5064	bass	1-6	-0.444444	1	-0.009208	0.2813324	-0.05	-0.216518	0.1031471
Score-dep.	N5480	bass	1-6	-0.333333	1	0.5144011	0.4071561	0.6201925	0.28125	0.833333
Score-dep.	N5484	bass	1-6	-0.4375	0.4	-0.215646	0.1799825	-0.25	-0.338542	-0.154762
Score-dep.	N5783	bass	1-6	-0.428571	0.5	-0.004049	0.2153177	0	-0.125	0.125
Score-dep.	N6128	bass	1-6	-0.3125	0.625	-0.015295	0.2698819	-0.090909	-0.202941	0.0555556
