# Supplementary material for: Behavioral Quantification of Audiomotor Transformations in Improvising and Score-Dependent Musicians
Source: PLoS One. 2016 Nov 11;11(11):e0166033. doi: 10.1371/journal.pone.0166033 (PMC5105996; doi:10.1371/journal.pone.0166033)
Supplement: S1 Alignment Scores — (ZIP) [file pone.0166033.s001.zip › Alignment_scores_2.pdf]

Alignment scores 2. IOR alignment: treble/bass.

GROUP	SUBJECT	VOICE	BLOCK	Min	Max	Mean	Stand. dev	Median	25 prcntil	75 prcntil
Improvising	N3851	treble	1-6	0.3125	0.890019	0.6417576	0.1784077	0.647865	0.497277	0.8180878
Improvising	N3933	treble	1-6	0.397108	0.917048	0.6442685	0.1423245	0.639492	0.516037	0.753633
Improvising	N3938	treble	1-6	0.183808	0.888531	0.594539	0.1908688	0.592585	0.418105	0.783405
Improvising	N3974	treble	1-6	0.167805	0.882335	0.5925162	0.1681791	0.5746745	0.4662573	0.7405763
Improvising	N4223	treble	1-6	0.220969	0.919378	0.6255611	0.1972232	0.675071	0.428561	0.770681
Improvising	N4229	treble	1-6	0.165711	0.875878	0.556214	0.1646878	0.518498	0.4490475	0.6994335
Improvising	N4258	treble	1-6	0.1	0.897828	0.5311305	0.2071834	0.547911	0.389963	0.686711
Improvising	N4486	treble	1-6	0.111111	0.785152	0.4654988	0.1645504	0.44045	0.341726	0.605753
Improvising	N4549	treble	1-6	0.287136	0.882909	0.603011	0.1587301	0.619229	0.4731355	0.7240125
Improvising	N4774	treble	1-6	0.301298	0.820654	0.5616074	0.1611608	0.553945	0.4546235	0.669077
Improvising	N4869	treble	1-6	0.327985	0.900558	0.6597127	0.1851887	0.705169	0.478955	0.828774
Improvising	N5692	treble	1-6	0.227357	0.888926	0.5613879	0.189753	0.505856	0.417571	0.7319335
Score-dep.	N4429	treble	1-6	0.182826	0.843865	0.4403639	0.1628903	0.398802	0.2959775	0.539825
Score-dep.	N4517	treble	1-6	0.200374	0.677532	0.4516619	0.1185427	0.462568	0.350625	0.5323495
Score-dep.	N4588	treble	1-6	0.190674	0.821535	0.4308522	0.1318641	0.42703	0.34102	0.506289
Score-dep.	N4615	treble	1-6	0.216372	0.920673	0.6223464	0.171023	0.636863	0.5118095	0.749644
Score-dep.	N4657	treble	1-6	0.0714286	0.569799	0.2828492	0.1202134	0.295218	0.2012242	0.3822345
Score-dep.	N5064	treble	1-6	0.2322	0.84106	0.4745976	0.1574633	0.416046	0.3334355	0.6213135
Score-dep.	N5480	treble	1-6	0.295046	0.909664	0.5494328	0.1908592	0.518326	0.38965	0.7193462
Score-dep.	N5484	treble	1-6	0.111111	0.802088	0.5024145	0.1907295	0.512033	0.349267	0.669781
Score-dep.	N5783	treble	1-6	0.270056	0.674897	0.4697135	0.1158195	0.452448	0.3752205	0.5755332
Score-dep.	N6128	treble	1-6	0.295255	0.844423	0.4748772	0.1253126	0.444473	0.3759505	0.530713

Alignment scores 2. IOR alignment: treble/bass.

GROUP	SUBJECT	VOICE	BLOCK	Min	Max	Mean	Stand. dev	Median	25 prcntil	75 prcntil
Improvising	N3851	bass	1-6	0.18809	0.799	0.4894953	0.1522126	0.488421	0.3628508	0.6078183
Improvising	N3933	bass	1-6	0.22006	0.8916	0.5149241	0.1701378	0.488694	0.376875	0.6222995
Improvising	N3938	bass	1-6	0.178055	0.90139	0.4831321	0.1978778	0.45355	0.3367685	0.5818965
Improvising	N3974	bass	1-6	0.125	0.832737	0.3821629	0.1445494	0.365167	0.2871937	0.4613862
Improvising	N4223	bass	1-6	0.214543	0.93873	0.505389	0.1751608	0.509591	0.3400905	0.622238
Improvising	N4229	bass	1-6	0.130493	0.934686	0.4667397	0.2078168	0.408834	0.3365555	0.5457425
Improvising	N4258	bass	1-6	0.111111	0.712617	0.3768923	0.1311723	0.40057	0.28706	0.4676815
Improvising	N4486	bass	1-6	0.111111	0.725544	0.3957553	0.1461341	0.39628	0.275233	0.466429
Improvising	N4549	bass	1-6	0.162727	0.85658	0.4492681	0.1463505	0.473502	0.3834345	0.512132
Improvising	N4774	bass	1-6	0.221975	0.815715	0.4523213	0.1529664	0.41418	0.3576325	0.5323145
Improvising	N4869	bass	1-6	0.0833333	0.87491	0.4504434	0.1925544	0.399914	0.3008645	0.554054
Improvising	N5692	bass	1-6	0.1	0.882617	0.5058198	0.1907562	0.454068	0.367698	0.6512095
Score-dep.	N4429	bass	1-6	0.1	0.787376	0.3689996	0.142919	0.349415	0.286501	0.441351
Score-dep.	N4517	bass	1-6	0.156666	0.684141	0.3757266	0.1423863	0.349444	0.254773	0.4601415
Score-dep.	N4588	bass	1-6	0.148868	0.538372	0.3145028	0.0970578	0.292564	0.2456393	0.3804495
Score-dep.	N4615	bass	1-6	0.111111	0.878335	0.4929138	0.1855024	0.471671	0.3688285	0.626065
Score-dep.	N4657	bass	1-6	0.0909091	0.43927	0.2149075	0.0949726	0.181301	0.1400715	0.286476
Score-dep.	N5064	bass	1-6	0.194073	0.734813	0.4146362	0.1374085	0.394723	0.305115	0.5086505
Score-dep.	N5480	bass	1-6	0.152611	0.880154	0.5078276	0.1932361	0.475321	0.3703017	0.6864095
Score-dep.	N5484	bass	1-6	0.0555556	0.39583	0.1811273	0.0883832	0.142857	0.111111	0.2409305
Score-dep.	N5783	bass	1-6	0.0978776	0.578298	0.3600175	0.1150229	0.371749	0.298187	0.423859
Score-dep.	N6128	bass	1-6	0.148362	0.625714	0.3449877	0.1115594	0.345334	0.268474	0.3997845
