# Supplementary material for: Behavioral Quantification of Audiomotor Transformations in Improvising and Score-Dependent Musicians
Source: PLoS One. 2016 Nov 11;11(11):e0166033. doi: 10.1371/journal.pone.0166033 (PMC5105996; doi:10.1371/journal.pone.0166033)
Supplement: S1 Alignment Scores — (ZIP) [file pone.0166033.s001.zip › Alignment_scores_3.pdf]

Alignment scores 3. Exact pitch treble alignment: replication/transposition.

GROUP	SUBJECT	VOICE	TASK	BLOCK	Min	Max	Mean	Stand. dev	Median	25 prcntil	75 prcntil
Improvising	N3851	treble	replication	3a/6a	0.5	1	0.78125	0.2477578	0.875	0.5	1
Improvising	N3933	treble	replication	3a/6a	0.458333	1	0.8297847	0.184745	0.830357	0.7548078	1
Improvising	N3938	treble	replication	3a/6a	0.125	1	0.7334572	0.3012187	0.780303	0.5788465	1
Improvising	N3974	treble	replication	3a/6a	-0.1875	1	0.6152645	0.4272492	0.7596155	0.2475962	0.9625
Improvising	N4223	treble	replication	3a/6a	0.464286	1	0.8072916	0.2168669	0.8541665	0.5714285	1
Improvising	N4229	treble	replication	3a/6a	0.384615	1	0.7498997	0.258996	0.8125	0.4765622	1
Improvising	N4258	treble	replication	3a/6a	-0.214286	1	0.6732143	0.4320101	0.8375	0.38125	1
Improvising	N4486	treble	replication	3a/6a	0	1	0.6927084	0.3550382	0.7589285	0.464286	1
Improvising	N4549	treble	replication	3a/6a	0.266667	1	0.6703526	0.2482373	0.6576925	0.5125	0.9326923
Improvising	N4774	treble	replication	3a/6a	0.4375	1	0.8719953	0.2385497	1	0.6538465	1
Improvising	N4869	treble	replication	3a/6a	0.3125	1	0.7771577	0.284878	0.9375	0.486607	1
Improvising	N5692	treble	replication	3a/6a	0.461538	1	0.7938191	0.1879617	0.815341	0.6374998	0.96875
Score-dep.	N4429	treble	replication	3a/6a	0.0789474	1	0.6075621	0.3161559	0.5747865	0.3823528	0.9423078
Score-dep.	N4517	treble	replication	3a/6a	0.230769	1	0.5704617	0.2956951	0.5588235	0.265625	0.8506945
Score-dep.	N4588	treble	replication	3a/6a	-0.03125	1	0.7166752	0.3443043	0.8541665	0.5600965	0.9732143
Score-dep.	N4615	treble	replication	3a/6a	0.678571	1	0.9285714	0.1336307	1	0.8125	1
Score-dep.	N4657	treble	replication	3a/6a	-0.038462	0.5	0.238397	0.1836871	0.235294	0.0950227	0.4178573
Score-dep.	N5064	treble	replication	3a/6a	0	1	0.5886729	0.3475397	0.615909	0.322917	0.9423078
Score-dep.	N5480	treble	replication	3a/6a	0.272727	1	0.808712	0.2619943	0.931818	0.6458333	1
Score-dep.	N5484	treble	replication	3a/6a	-0.1	1	0.703612	0.3749923	0.834091	0.4964285	0.96875
Score-dep.	N5783	treble	replication	3a/6a	0.133333	1	0.5774925	0.3438552	0.5	0.2734375	0.9732143
Score-dep.	N6128	treble	replication	3a/6a	0.115385	1	0.5925481	0.3222635	0.625	0.2500003	0.8645833

Alignment scores 3. Exact pitch treble alignment: replication/transposition.

GROUP	SUBJECT	VOICE	TASK	BLOCK	Min	Max	Mean	Stand. dev	Median	25 prcntil	75 prcntil
Improvising	N3851	treble	transposition	3b/6b	0.346154	1	0.7126069	0.2722398	0.715812	0.4423078	1
Improvising	N3933	treble	transposition	3b/6b	0	1	0.5452599	0.404666	0.748252	0.0980903	0.8526785
Improvising	N3938	treble	transposition	3b/6b	0.2	1	0.515792	0.3105007	0.4090905	0.3063913	0.8653845
Improvising	N3974	treble	transposition	3b/6b	0	1	0.4227461	0.3629048	0.3846155	0.1144483	0.7269233
Improvising	N4223	treble	transposition	3b/6b	-0.083333	1	0.5943948	0.3440126	0.638889	0.425	0.8526785
Improvising	N4229	treble	transposition	3b/6b	0	1	0.6339984	0.3309436	0.5996505	0.4875565	0.965909
Improvising	N4258	treble	transposition	3b/6b	-0.1	1	0.5533854	0.4763633	0.7135415	0.025	1
Improvising	N4486	treble	transposition	3b/6b	-0.25	0.833333	0.3398989	0.3946991	0.3484845	-0.011111	0.7375002
Improvising	N4549	treble	transposition	3b/6b	-0.25	1	0.2362837	0.4303816	0.1756408	-0.129688	0.532143
Improvising	N4774	treble	transposition	3b/6b	0	1	0.4927917	0.3870254	0.397619	0.1832385	0.9444445
Improvising	N4869	treble	transposition	3b/6b	-0.416667	1	0.4808036	0.5449991	0.5982145	-0.033333	1
Improvising	N5692	treble	transposition	3b/6b	0.136364	1	0.7163803	0.2793682	0.788889	0.591346	0.9545455
Score-dep.	N4429	treble	transposition	3b/6b	-0.153846	0.71875	0.1635818	0.2682326	0.143029	-0.055769	0.2451923
Score-dep.	N4517	treble	transposition	3b/6b	-0.166667	0.708333	0.1360843	0.2977126	0.1099627	-0.125	0.3161458
Score-dep.	N4588	treble	transposition	3b/6b	-0.055556	0.75	0.3013775	0.2754641	0.2708335	0.0913462	0.565476
Score-dep.	N4615	treble	transposition	3b/6b	0	1	0.6113592	0.372425	0.763889	0.2053572	0.8645833
Score-dep.	N4657	treble	transposition	3b/6b	-0.392857	0.0357143	-0.213795	0.15247	-0.238889	-0.350852	-0.072552
Score-dep.	N5064	treble	transposition	3b/6b	0.0909091	0.5625	0.3244724	0.1518338	0.3535715	0.1806818	0.421875
Score-dep.	N5480	treble	transposition	3b/6b	0.0909091	1	0.7409554	0.3620189	0.863636	0.375	1
Score-dep.	N5484	treble	transposition	3b/6b	-0.416667	1	0.3566326	0.523924	0.321429	-0.035714	0.8
Score-dep.	N5783	treble	transposition	3b/6b	-0.088235	0.9	0.319502	0.4003494	0.34375	-0.083333	0.777778
Score-dep.	N6128	treble	transposition	3b/6b	-0.1	0.611111	0.2081597	0.2488537	0.1604165	-0.010417	0.4479167
