# Supplementary material for: Behavioral Quantification of Audiomotor Transformations in Improvising and Score-Dependent Musicians
Source: PLoS One. 2016 Nov 11;11(11):e0166033. doi: 10.1371/journal.pone.0166033 (PMC5105996; doi:10.1371/journal.pone.0166033)
Supplement: S1 Alignment Scores — (ZIP) [file pone.0166033.s001.zip › Alignment_scores_4.pdf]

Alignment scores 4. IOR treble alignment: replication/transposition.

GROUP	SUBJECT	VOICE	TASK	BLOCK	Min	Max	Mean	Stand. dev	Median	25 prcntil	75 prcntil
Improvising	N3851	treble	replication	3a/6a	0.47064	0.866536	0.6774745	0.1703188	0.716311	0.4953687	0.8398737
Improvising	N3933	treble	replication	3a/6a	0.397108	0.917048	0.7124404	0.168082	0.7275415	0.6172267	0.8532492
Improvising	N3938	treble	replication	3a/6a	0.362336	0.888531	0.6734552	0.1996561	0.7369805	0.460336	0.848734
Improvising	N3974	treble	replication	3a/6a	0.458999	0.849893	0.6107043	0.1461221	0.592159	0.4726557	0.7503553
Improvising	N4223	treble	replication	3a/6a	0.362338	0.880784	0.6384434	0.1781332	0.676266	0.4550623	0.769006
Improvising	N4229	treble	replication	3a/6a	0.466026	0.875878	0.6485869	0.1627346	0.5662095	0.5269975	0.8311925
Improvising	N4258	treble	replication	3a/6a	0.422433	0.897828	0.6043416	0.1793532	0.5278035	0.4577028	0.776842
Improvising	N4486	treble	replication	3a/6a	0.319627	0.750071	0.5683835	0.1634494	0.585111	0.432477	0.7257485
Improvising	N4549	treble	replication	3a/6a	0.348853	0.787602	0.6233945	0.1373708	0.628322	0.5499095	0.7251483
Improvising	N4774	treble	replication	3a/6a	0.449756	0.820654	0.6633251	0.15471	0.714632	0.4873565	0.7995383
Improvising	N4869	treble	replication	3a/6a	0.379152	0.878837	0.6673233	0.2042136	0.7554415	0.4304658	0.8397653
Improvising	N5692	treble	replication	3a/6a	0.424972	0.865141	0.631136	0.1776445	0.639999	0.445145	0.7993623
Score-dep.	N4429	treble	replication	3a/6a	0.354642	0.827323	0.5683581	0.1605218	0.581886	0.4064707	0.6718142
Score-dep.	N4517	treble	replication	3a/6a	0.441981	0.658397	0.5186783	0.0832918	0.4786685	0.462693	0.6084812
Score-dep.	N4588	treble	replication	3a/6a	0.34102	0.821535	0.5019455	0.1478614	0.4651745	0.4081522	0.5628893
Score-dep.	N4615	treble	replication	3a/6a	0.58987	0.774275	0.6775324	0.072291	0.6628925	0.6133852	0.753318
Score-dep.	N4657	treble	replication	3a/6a	0.33341	0.466455	0.4005525	0.0455058	0.3907295	0.3638905	0.44216
Score-dep.	N5064	treble	replication	3a/6a	0.305045	0.84106	0.5202479	0.2033858	0.491186	0.323027	0.6978825
Score-dep.	N5480	treble	replication	3a/6a	0.295046	0.909664	0.618614	0.2475828	0.658026	0.3699223	0.8463363
Score-dep.	N5484	treble	replication	3a/6a	0.129969	0.802088	0.5435761	0.1988021	0.574917	0.4787868	0.6645243
Score-dep.	N5783	treble	replication	3a/6a	0.316843	0.643231	0.4796581	0.10754	0.4799645	0.3971378	0.5631928
Score-dep.	N6128	treble	replication	3a/6a	0.332905	0.844423	0.515373	0.161824	0.45866	0.4121188	0.6050443

Alignment scores 4. IOR treble alignment: replication/transposition.

GROUP	SUBJECT	VOICE	TASK	BLOCK	Min	Max	Mean	Stand. dev	Median	25 prcntil	75 prcntil
Improvising	N3851	treble	transposition	3b/6b	0.507053	0.890019	0.7135869	0.1647332	0.7290455	0.5430782	0.8684177
Improvising	N3933	treble	transposition	3b/6b	0.413638	0.836056	0.6465399	0.1355445	0.6655225	0.5337922	0.7422703
Improvising	N3938	treble	transposition	3b/6b	0.367821	0.813331	0.608719	0.1632105	0.5593725	0.5048442	0.79294
Improvising	N3974	treble	transposition	3b/6b	0.465972	0.815105	0.5904209	0.1373809	0.561991	0.468403	0.731603
Improvising	N4223	treble	transposition	3b/6b	0.592458	0.919378	0.7425406	0.1213043	0.7336665	0.619631	0.8660245
Improvising	N4229	treble	transposition	3b/6b	0.439888	0.759456	0.5803155	0.1201278	0.5251965	0.488513	0.7053198
Improvising	N4258	treble	transposition	3b/6b	0.35347	0.87384	0.6470096	0.178112	0.669187	0.4964507	0.815782
Improvising	N4486	treble	transposition	3b/6b	0.302071	0.657176	0.4652579	0.1142704	0.45496	0.3645435	0.545485
Improvising	N4549	treble	transposition	3b/6b	0.359909	0.773497	0.5848256	0.1239811	0.6066465	0.504304	0.664033
Improvising	N4774	treble	transposition	3b/6b	0.309989	0.800327	0.5726357	0.1854284	0.5977325	0.3824893	0.759835
Improvising	N4869	treble	transposition	3b/6b	0.347004	0.889633	0.7241772	0.1892993	0.8048095	0.579903	0.832766
Improvising	N5692	treble	transposition	3b/6b	0.377032	0.888926	0.6362341	0.2164428	0.58548	0.4430545	0.8719347
Score-dep.	N4429	treble	transposition	3b/6b	0.25744	0.650685	0.4793901	0.1295936	0.4620485	0.4016888	0.612458
Score-dep.	N4517	treble	transposition	3b/6b	0.331753	0.615298	0.4719724	0.1021508	0.464985	0.3785428	0.5764113
Score-dep.	N4588	treble	transposition	3b/6b	0.190674	0.604961	0.4146384	0.1244239	0.406273	0.3449255	0.5074495
Score-dep.	N4615	treble	transposition	3b/6b	0.332054	0.920673	0.663935	0.2008694	0.720307	0.4728067	0.8147215
Score-dep.	N4657	treble	transposition	3b/6b	0.0714286	0.322393	0.1813868	0.0827783	0.1956145	0.0994318	0.2320907
Score-dep.	N5064	treble	transposition	3b/6b	0.322835	0.679223	0.4182445	0.1182173	0.3748445	0.333553	0.4644618
Score-dep.	N5480	treble	transposition	3b/6b	0.338562	0.844726	0.6498584	0.2110324	0.692947	0.38651	0.824707
Score-dep.	N5484	treble	transposition	3b/6b	0.307837	0.764448	0.5647759	0.1474893	0.572301	0.488438	0.693815
Score-dep.	N5783	treble	transposition	3b/6b	0.380724	0.654436	0.5077684	0.107905	0.51066	0.391053	0.62397
Score-dep.	N6128	treble	transposition	3b/6b	0.364467	0.771897	0.496741	0.1248349	0.45789	0.4288325	0.5385885
