# Supplementary material for: Behavioral Quantification of Audiomotor Transformations in Improvising and Score-Dependent Musicians
Source: PLoS One. 2016 Nov 11;11(11):e0166033. doi: 10.1371/journal.pone.0166033 (PMC5105996; doi:10.1371/journal.pone.0166033)
Supplement: S1 Alignment Scores — (ZIP) [file pone.0166033.s001.zip › Alignment_scores_5.pdf]

Alignment scores 5. Exact pitch bass alignment: replication/transposition.

GROUP	SUBJECT	VOICE	TASK	BLOCK	Min	Max	Mean	Stand. dev	Median	25 prcntil	75 prcntil
Improvising	N3851	bass	replication	3a/6a	-0.25	1	0.3398336	0.4141403	0.299107	0.0113636	0.68125
Improvising	N3933	bass	replication	3a/6a	-0.181818	1	0.318263	0.3717001	0.288961	0.03125	0.56875
Improvising	N3938	bass	replication	3a/6a	-0.642857	1	0.1038961	0.4893019	0.0227273	-0.214286	0.3928567
Improvising	N3974	bass	replication	3a/6a	-0.571429	1	0.0421875	0.5107839	-0.05	-0.390625	0.3928571
Improvising	N4223	bass	replication	3a/6a	-0.277778	0.7	0.3061778	0.3262749	0.3253965	0.0876624	0.6125002
Improvising	N4229	bass	replication	3a/6a	0.1875	1	0.6065902	0.3052601	0.472222	0.428571	0.965909
Improvising	N4258	bass	replication	3a/6a	-0.3125	0.4	-0.070982	0.2168485	-0.102679	-0.225	-0.0125
Improvising	N4486	bass	replication	3a/6a	-0.277778	1	0.0836309	0.400385	-0.027778	-0.181548	0.15
Improvising	N4549	bass	replication	3a/6a	-0.444444	0.35	-0.0125	0.2860195	-0.027778	-0.265625	0.271875
Improvising	N4774	bass	replication	3a/6a	-0.1875	1	0.2887153	0.3565296	0.2333335	0.0666667	0.46875
Improvising	N4869	bass	replication	3a/6a	-0.5	1	0.101425	0.5794776	0.0636365	-0.486111	0.6321427
Improvising	N5692	bass	replication	3a/6a	-0.357143	1	0.6032738	0.5261696	0.8416665	0.1	1
Score-dep.	N4429	bass	replication	3a/6a	-0.307692	1	0.0243541	0.4181911	-0.123738	-0.223214	0.1071428
Score-dep.	N4517	bass	replication	3a/6a	-0.4	0.714286	0.1194197	0.3249586	0.1875	-0.092857	0.234375
Score-dep.	N4588	bass	replication	3a/6a	-0.125	0.785714	0.1866743	0.2808162	0.1512606	-0.02381	0.2785715
Score-dep.	N4615	bass	replication	3a/6a	-0.090909	1	0.4014341	0.3857275	0.3482145	0.10625	0.7023812
Score-dep.	N4657	bass	replication	3a/6a	-0.5625	0.625	-0.121875	0.3527159	-0.1875	-0.325	0.015625
Score-dep.	N5064	bass	replication	3a/6a	-0.272727	0.428571	-0.048368	0.2254964	-0.089286	-0.229167	0.0585665
Score-dep.	N5480	bass	replication	3a/6a	0.571429	1	0.843006	0.1838212	0.9166665	0.6473215	1
Score-dep.	N5484	bass	replication	3a/6a	-0.4375	0.4	-0.237277	0.2882531	-0.366072	-0.428571	-0.103571
Score-dep.	N5783	bass	replication	3a/6a	-0.25	0.375	0.0247227	0.1822676	0	-0.078599	0.1227678
Score-dep.	N6128	bass	replication	3a/6a	-0.277778	0.625	0.0387153	0.3553195	-0.08125	-0.234375	0.421875

Alignment scores 5. Exact pitch bass alignment: replication/transposition.

GROUP	SUBJECT	VOICE	TASK	BLOCK	Min	Max	Mean	Stand. dev	Median	25 prcntil	75 prcntil
Improvising	N3851	bass	transposition	3b/6b	-0.357143	1	0.2512378	0.4677736	0.075	-0.058239	0.68125
Improvising	N3933	bass	transposition	3b/6b	-0.142857	1	0.155063	0.3935862	0.0051282	-0.136364	0.3281247
Improvising	N3938	bass	transposition	3b/6b	-0.571429	1	0.0307539	0.4818456	-0.072917	-0.262153	0.3110116
Improvising	N3974	bass	transposition	3b/6b	-0.642857	0.125	-0.16942	0.2685237	-0.08125	-0.421875	0.034375
Improvising	N4223	bass	transposition	3b/6b	-0.571429	0.8	0.1894096	0.4793996	0.2276785	-0.197917	0.6696427
Improvising	N4229	bass	transposition	3b/6b	-0.3125	1	0.6004464	0.4423474	0.625	0.4308032	1
Improvising	N4258	bass	transposition	3b/6b	-0.357143	0.214286	-0.089534	0.1607833	-0.1125	-0.148214	-0.013889
Improvising	N4486	bass	transposition	3b/6b	-0.25	0.107143	-0.119072	0.1159986	-0.125	-0.21875	-0.063542
Improvising	N4549	bass	transposition	3b/6b	-0.4375	0	-0.157907	0.1371058	-0.125	-0.238636	-0.069444
Improvising	N4774	bass	transposition	3b/6b	-0.1875	0.666667	0.0856227	0.3137562	-0.038462	-0.171875	0.363095
Improvising	N4869	bass	transposition	3b/6b	-0.714286	0.111111	-0.188425	0.2354777	-0.139611	-0.225	-0.10625
Improvising	N5692	bass	transposition	3b/6b	-0.1	1	0.4730769	0.4665626	0.3798075	0.03125	1
Score-dep.	N4429	bass	transposition	3b/6b	-0.642857	0.15	-0.21713	0.2644447	-0.275253	-0.358173	0.0686813
Score-dep.	N4517	bass	transposition	3b/6b	-0.3125	0.4375	-0.063402	0.2404363	-0.133929	-0.206169	0.079365
Score-dep.	N4588	bass	transposition	3b/6b	-0.5	0.0555556	-0.227059	0.1651339	-0.21875	-0.339286	-0.135417
Score-dep.	N4615	bass	transposition	3b/6b	-0.15	0.785714	0.1638144	0.299569	0.09375	-0.041667	0.3357142
Score-dep.	N4657	bass	transposition	3b/6b	-0.375	0.0625	-0.234821	0.14516	-0.285714	-0.345982	-0.14375
Score-dep.	N5064	bass	transposition	3b/6b	-0.444444	0	-0.18941	0.1277235	-0.190477	-0.22601	-0.117789
Score-dep.	N5480	bass	transposition	3b/6b	0.428571	1	0.7390229	0.2277082	0.714286	0.5	1
Score-dep.	N5484	bass	transposition	3b/6b	-0.35	-0.071429	-0.213265	0.1055781	-0.25	-0.285714	-0.071429
Score-dep.	N5783	bass	transposition	3b/6b	-0.25	0.375	0.0876984	0.2233027	0.1	-0.111111	0.285714
Score-dep.	N6128	bass	transposition	3b/6b	-0.277778	0.4	-0.019523	0.2181375	-0.045455	-0.182292	0.1250003
