# Supplementary material for: Behavioral Quantification of Audiomotor Transformations in Improvising and Score-Dependent Musicians
Source: PLoS One. 2016 Nov 11;11(11):e0166033. doi: 10.1371/journal.pone.0166033 (PMC5105996; doi:10.1371/journal.pone.0166033)
Supplement: S1 Alignment Scores — (ZIP) [file pone.0166033.s001.zip › Alignment_scores_6.pdf]

Alignment scores 6. IOR bass alignment: replication/transposition.

GROUP	SUBJECT	VOICE	TASK	BLOCK	Min	Max	Mean	Stand. dev	Median	25 prcntil	75 prcntil
Improvising	N3851	bass	replication	3a/6a	0.263001	0.799	0.5618857	0.1754453	0.5990645	0.405257	0.681666
Improvising	N3933	bass	replication	3a/6a	0.361234	0.764773	0.5360207	0.1621726	0.510486	0.3726542	0.698494
Improvising	N3938	bass	replication	3a/6a	0.295121	0.90139	0.4939893	0.220141	0.420043	0.3334928	0.6854555
Improvising	N3974	bass	replication	3a/6a	0.220836	0.5425	0.3856886	0.0955728	0.382521	0.3335383	0.45334
Improvising	N4223	bass	replication	3a/6a	0.316048	0.646234	0.4388516	0.146534	0.3590515	0.3201722	0.6183947
Improvising	N4229	bass	replication	3a/6a	0.363721	0.867548	0.6014389	0.2028097	0.513627	0.4351137	0.8353097
Improvising	N4258	bass	replication	3a/6a	0.205095	0.712617	0.4360571	0.1423479	0.425156	0.3685263	0.4820575
Improvising	N4486	bass	replication	3a/6a	0.226248	0.725544	0.4134649	0.155068	0.389481	0.2909018	0.4852545
Improvising	N4549	bass	replication	3a/6a	0.172533	0.85658	0.4778164	0.1928811	0.498248	0.3649185	0.5150715
Improvising	N4774	bass	replication	3a/6a	0.245046	0.731656	0.4463981	0.1710674	0.4209145	0.3000938	0.6023983
Improvising	N4869	bass	replication	3a/6a	0.2528	0.848215	0.4873514	0.2193486	0.3948185	0.3143898	0.7156045
Improvising	N5692	bass	replication	3a/6a	0.303527	0.82906	0.5906464	0.1978888	0.6032505	0.3816225	0.7879458
Score-dep.	N4429	bass	replication	3a/6a	0.270305	0.787376	0.4652986	0.1481282	0.446109	0.3875525	0.489636
Score-dep.	N4517	bass	replication	3a/6a	0.247579	0.684141	0.4599684	0.1633813	0.416282	0.3080087	0.6271135
Score-dep.	N4588	bass	replication	3a/6a	0.293332	0.538372	0.3918049	0.093289	0.3592915	0.3164143	0.4944752
Score-dep.	N4615	bass	replication	3a/6a	0.271197	0.63613	0.4469817	0.1255179	0.4389495	0.325524	0.553403
Score-dep.	N4657	bass	replication	3a/6a	0.111111	0.43927	0.2408241	0.1086287	0.20882	0.16421	0.329594
Score-dep.	N5064	bass	replication	3a/6a	0.20806	0.67231	0.389788	0.1625466	0.36387	0.2319865	0.5256195
Score-dep.	N5480	bass	replication	3a/6a	0.326998	0.868265	0.6385376	0.1874122	0.6664425	0.482222	0.7957943
Score-dep.	N5484	bass	replication	3a/6a	0.0909091	0.285585	0.1743591	0.0709677	0.1637635	0.1145832	0.2362652
Score-dep.	N5783	bass	replication	3a/6a	0.259147	0.539964	0.3744881	0.0966568	0.3964325	0.2713592	0.4251835
Score-dep.	N6128	bass	replication	3a/6a	0.292666	0.581707	0.3847194	0.1098256	0.345749	0.3088982	0.4897523

Alignment scores 6. IOR bass alignment: replication/transposition.

GROUP	SUBJECT	VOICE	TASK	BLOCK	Min	Max	Mean	Stand. dev	Median	25 prcntil	75 prcntil
Improvising	N3851	bass	transposition	3b/6b	0.277768	0.755977	0.5049794	0.1492661	0.519959	0.3800458	0.6039897
Improvising	N3933	bass	transposition	3b/6b	0.22006	0.822255	0.5022587	0.2062761	0.4864045	0.3062158	0.6736598
Improvising	N3938	bass	transposition	3b/6b	0.348302	0.819253	0.5390111	0.1758069	0.4906465	0.3799112	0.7226805
Improvising	N3974	bass	transposition	3b/6b	0.189483	0.547429	0.3356276	0.1079076	0.335096	0.25265	0.3798002
Improvising	N4223	bass	transposition	3b/6b	0.332658	0.873097	0.578449	0.2056185	0.5631915	0.3663192	0.7915313
Improvising	N4229	bass	transposition	3b/6b	0.258828	0.851977	0.4990704	0.1783158	0.487752	0.3825885	0.5729135
Improvising	N4258	bass	transposition	3b/6b	0.198812	0.498258	0.3977294	0.1055992	0.434915	0.3068585	0.4713702
Improvising	N4486	bass	transposition	3b/6b	0.237244	0.690413	0.3868584	0.1866412	0.28501	0.2472628	0.5968058
Improvising	N4549	bass	transposition	3b/6b	0.162727	0.653892	0.4491019	0.1713106	0.475564	0.2953888	0.5963173
Improvising	N4774	bass	transposition	3b/6b	0.292727	0.815715	0.4655446	0.1638302	0.4101625	0.3699635	0.54799
Improvising	N4869	bass	transposition	3b/6b	0.29994	0.87491	0.4818992	0.1951386	0.396178	0.360774	0.6257885
Improvising	N5692	bass	transposition	3b/6b	0.264162	0.882617	0.5954666	0.2246869	0.547218	0.434799	0.830396
Score-dep.	N4429	bass	transposition	3b/6b	0.254547	0.436843	0.3331741	0.0593192	0.325989	0.288921	0.3767218
Score-dep.	N4517	bass	transposition	3b/6b	0.341833	0.636193	0.454258	0.1011691	0.447276	0.3567035	0.5292665
Score-dep.	N4588	bass	transposition	3b/6b	0.170041	0.410995	0.3008326	0.0937645	0.3151145	0.2083015	0.3882705
Score-dep.	N4615	bass	transposition	3b/6b	0.266214	0.828698	0.5252289	0.2187441	0.4515735	0.3372645	0.7804095
Score-dep.	N4657	bass	transposition	3b/6b	0.0909091	0.25	0.15666	0.0565447	0.142857	0.1145832	0.2143305
Score-dep.	N5064	bass	transposition	3b/6b	0.194073	0.625194	0.4111616	0.1680494	0.3979185	0.2446888	0.591562
Score-dep.	N5480	bass	transposition	3b/6b	0.373882	0.880154	0.5991614	0.1757445	0.562283	0.464155	0.712329
Score-dep.	N5484	bass	transposition	3b/6b	0.1	0.344962	0.182299	0.0928391	0.142857	0.1	0.263274
Score-dep.	N5783	bass	transposition	3b/6b	0.252131	0.423859	0.3646174	0.0621834	0.38642	0.310089	0.412391
Score-dep.	N6128	bass	transposition	3b/6b	0.217604	0.625714	0.3341396	0.1295771	0.29577	0.2494823	0.3689005
