# Supplementary material for: Behavioral Quantification of Audiomotor Transformations in Improvising and Score-Dependent Musicians
Source: PLoS One. 2016 Nov 11;11(11):e0166033. doi: 10.1371/journal.pone.0166033 (PMC5105996; doi:10.1371/journal.pone.0166033)
Supplement: S1 Alignment Scores — (ZIP) [file pone.0166033.s001.zip › Alignment_scores_7.pdf]

Alignment scores 7. Exact pitch treble alignment: feedback/no feedback.

GROUP	SUBJECT	VOICE	CONDITION	BLOCKS	Min	Max	Mean	Stand. dev	Median	25 prcntil	75 prcntil
Improvising	N3851	treble	feedback	3a, 4, 5, 6a	0	1	0.7399268	0.2989986	0.761905	0.5	1
Improvising	N3933	treble	feedback	3a, 4, 5, 6a	0.34375	1	0.7594306	0.2154841	0.7774725	0.575	1
Improvising	N3938	treble	feedback	3a, 4, 5, 6a	-0.211538	1	0.5373608	0.3910506	0.599359	0.1676135	0.8749997
Improvising	N3974	treble	feedback	3a, 4, 5, 6a	-0.285714	1	0.5023261	0.4669817	0.6826925	0.0643383	0.8928573
Improvising	N4223	treble	feedback	3a, 4, 5, 6a	-0.125	1	0.6448979	0.3724036	0.7761905	0.473214	1
Improvising	N4229	treble	feedback	3a, 4, 5, 6a	0.214286	1	0.7112636	0.2739686	0.7083335	0.4540437	1
Improvising	N4258	treble	feedback	3a, 4, 5, 6a	-0.214286	1	0.6077246	0.3760365	0.7125	0.2875	0.925
Improvising	N4486	treble	feedback	3a, 4, 5, 6a	0	1	0.4896374	0.3679371	0.416667	0.1936093	0.90625
Improvising	N4549	treble	feedback	3a, 4, 5, 6a	-0.159091	1	0.4839476	0.3383503	0.5477275	0.1738725	0.7076923
Improvising	N4774	treble	feedback	3a, 4, 5, 6a	0.0555556	1	0.719313	0.3179465	0.8487395	0.4665175	1
Improvising	N4869	treble	feedback	3a, 4, 5, 6a	-0.025	1	0.6633129	0.3483677	0.797059	0.4218747	1
Improvising	N5692	treble	feedback	3a, 4, 5, 6a	0.263158	1	0.6565491	0.2636995	0.75	0.3906253	0.8616073
Score-dep.	N4429	treble	feedback	3a, 4, 5, 6a	-0.375	1	0.4025567	0.4228462	0.5245215	0.1864033	0.650641
Score-dep.	N4517	treble	feedback	3a, 4, 5, 6a	-0.1	1	0.4170225	0.3371464	0.28125	0.1614585	0.7694448
Score-dep.	N4588	treble	feedback	3a, 4, 5, 6a	-0.222222	1	0.4970647	0.4507568	0.581731	-0.042188	0.8794643
Score-dep.	N4615	treble	feedback	3a, 4, 5, 6a	-0.15625	1	0.712432	0.3864461	0.9285715	0.366848	1
Score-dep.	N4657	treble	feedback	3a, 4, 5, 6a	-0.130435	0.5	0.1465655	0.1873857	0.1053116	0.0138221	0.2788868
Score-dep.	N5064	treble	feedback	3a, 4, 5, 6a	-0.086957	1	0.4298899	0.3923056	0.416667	-0.0125	0.791209
Score-dep.	N5480	treble	feedback	3a, 4, 5, 6a	-0.1875	1	0.6339889	0.3658307	0.6979165	0.3025567	1
Score-dep.	N5484	treble	feedback	3a, 4, 5, 6a	-0.1	1	0.6824313	0.3129013	0.785714	0.526923	0.875
Score-dep.	N5783	treble	feedback	3a, 4, 5, 6a	-0.1	1	0.4021174	0.3569731	0.3483455	0.1083331	0.5982143
Score-dep.	N6128	treble	feedback	3a, 4, 5, 6a	-0.263158	1	0.3929827	0.3654805	0.320707	0.1538465	0.7083335

Alignment scores 7. Exact pitch treble alignment: feedback/no feedback.

GROUP	SUBJECT	VOICE	CONDITION	BLOCKS	Min	Max	Mean	Stand. dev	Median	25 prcntil	75 prcntil
Improvising	N3851	treble	no feedback	1,2	-0.090909	1	0.6686889	0.3030185	0.666667	0.571429	0.863636
Improvising	N3933	treble	no feedback	1,2	0.142857	1	0.6935428	0.2518635	0.769231	0.535714	0.85
Improvising	N3938	treble	no feedback	1,2	-0.214286	1	0.4512283	0.4170496	0.375	0.166667	0.833333
Improvising	N3974	treble	no feedback	1,2	-0.214286	1	0.570982	0.4073349	0.6979165	0.1997768	0.8749997
Improvising	N4223	treble	no feedback	1,2	-0.357143	1	0.3739571	0.3950781	0.35	0.142857	0.590909
Improvising	N4229	treble	no feedback	1,2	0	1	0.4864036	0.3616906	0.5625	0.117647	0.8125
Improvising	N4258	treble	no feedback	1,2	-0.35	1	0.3555226	0.4385255	0.375	0.142857	0.8125
Improvising	N4486	treble	no feedback	1,2	-0.125	0.884615	0.2452844	0.2858503	0.153846	0.0714286	0.388889
Improvising	N4549	treble	no feedback	1,2	-0.038462	1	0.3530608	0.3318941	0.222222	0.115385	0.5
Improvising	N4774	treble	no feedback	1,2	0.375	1	0.6891335	0.211265	0.7	0.55	0.8
Improvising	N4869	treble	no feedback	1,2	-0.05	1	0.4007284	0.3608391	0.285714	0.142857	0.653846
Improvising	N5692	treble	no feedback	1,2	0.142857	1	0.677863	0.2915874	0.8125	0.5	0.875
Score-dep.	N4429	treble	no feedback	1,2	-0.416667	0.8	0.1093802	0.4205319	0.1875	-0.3	0.5
Score-dep.	N4517	treble	no feedback	1,2	-0.133333	1	0.3579015	0.3330383	0.333333	0.0625	0.5
Score-dep.	N4588	treble	no feedback	1,2	-0.088235	0.863636	0.3423064	0.3279931	0.25	0.05	0.6339285
Score-dep.	N4615	treble	no feedback	1,2	0.125	1	0.6672785	0.3421	0.833333	0.3125	1
Score-dep.	N4657	treble	no feedback	1,2	-0.35	0.375	-0.064335	0.2603774	-0.111111	-0.297619	0.1369792
Score-dep.	N5064	treble	no feedback	1,2	-0.071429	0.714286	0.4243374	0.261316	0.5	0.2	0.625
Score-dep.	N5480	treble	no feedback	1,2	-0.125	1	0.4828944	0.3294095	0.55	0.416667	0.681818
Score-dep.	N5484	treble	no feedback	1,2	-0.142857	1	0.4924193	0.4187645	0.5	0.0714286	0.863636
Score-dep.	N5783	treble	no feedback	1,2	-0.2	0.785714	0.166372	0.3199411	0.0714286	-0.090909	0.40625
Score-dep.	N6128	treble	no feedback	1,2	-0.222222	1	0.2303297	0.3685749	0.192308	-0.071429	0.5
