# Supplementary material for: Behavioral Quantification of Audiomotor Transformations in Improvising and Score-Dependent Musicians
Source: PLoS One. 2016 Nov 11;11(11):e0166033. doi: 10.1371/journal.pone.0166033 (PMC5105996; doi:10.1371/journal.pone.0166033)
Supplement: S1 Alignment Scores — (ZIP) [file pone.0166033.s001.zip › Alignment_scores_8.pdf]

Alignment scores 8. IOR treble alignment: feedback/no feedback.

GROUP	SUBJECT	VOICE	CONDITION	BLOCKS	Min	Max	Mean	Stand. dev	Median	25 prcntil	75 prcntil
Improvising	N3851	treble	feedback	3a, 4, 5, 6a	0.315877	0.866536	0.6840382	0.1816605	0.781841	0.4958305	0.835279
Improvising	N3933	treble	feedback	3a, 4, 5, 6a	0.397108	0.917048	0.6979574	0.1564623	0.706464	0.6187023	0.8396805
Improvising	N3938	treble	feedback	3a, 4, 5, 6a	0.362336	0.888531	0.6575346	0.1806897	0.6772295	0.455562	0.8074328
Improvising	N3974	treble	feedback	3a, 4, 5, 6a	0.364162	0.882335	0.6251224	0.1666467	0.592159	0.4911593	0.7952285
Improvising	N4223	treble	feedback	3a, 4, 5, 6a	0.351343	0.880784	0.6320541	0.1819706	0.676266	0.4131323	0.7774685
Improvising	N4229	treble	feedback	3a, 4, 5, 6a	0.393959	0.875878	0.6147436	0.1749315	0.5583355	0.4640712	0.8159695
Improvising	N4258	treble	feedback	3a, 4, 5, 6a	0.407643	0.897828	0.5929404	0.1581277	0.5533425	0.4573738	0.753286
Improvising	N4486	treble	feedback	3a, 4, 5, 6a	0.25107	0.750071	0.5013248	0.1709404	0.4728685	0.3446838	0.6745678
Improvising	N4549	treble	feedback	3a, 4, 5, 6a	0.28975	0.787602	0.588903	0.1497251	0.6281625	0.4950952	0.7228767
Improvising	N4774	treble	feedback	3a, 4, 5, 6a	0.331133	0.820654	0.5935279	0.1711897	0.5544075	0.461078	0.774285
Improvising	N4869	treble	feedback	3a, 4, 5, 6a	0.379152	0.878837	0.674811	0.1919608	0.7554415	0.453225	0.8526353
Improvising	N5692	treble	feedback	3a, 4, 5, 6a	0.424972	0.865141	0.6000097	0.1579523	0.540082	0.446591	0.7878787
Score-dep.	N4429	treble	feedback	3a, 4, 5, 6a	0.276647	0.827323	0.4851659	0.1627631	0.457701	0.370131	0.641443
Score-dep.	N4517	treble	feedback	3a, 4, 5, 6a	0.321443	0.658397	0.4667831	0.0995227	0.462818	0.3907932	0.528109
Score-dep.	N4588	treble	feedback	3a, 4, 5, 6a	0.267776	0.821535	0.4648516	0.136942	0.462065	0.3615483	0.5196102
Score-dep.	N4615	treble	feedback	3a, 4, 5, 6a	0.366851	0.856069	0.651767	0.1207638	0.6439575	0.605047	0.743682
Score-dep.	N4657	treble	feedback	3a, 4, 5, 6a	0.247322	0.466455	0.3583849	0.0684741	0.370658	0.3082632	0.4055532
Score-dep.	N5064	treble	feedback	3a, 4, 5, 6a	0.305045	0.84106	0.4839686	0.1950361	0.411443	0.3254445	0.6862355
Score-dep.	N5480	treble	feedback	3a, 4, 5, 6a	0.295046	0.909664	0.5517359	0.2283366	0.508646	0.3242345	0.7901193
Score-dep.	N5484	treble	feedback	3a, 4, 5, 6a	0.129969	0.802088	0.5576554	0.1664156	0.532161	0.4801245	0.661874
Score-dep.	N5783	treble	feedback	3a, 4, 5, 6a	0.316843	0.643231	0.4833546	0.0997284	0.4792745	0.4155992	0.5755332
Score-dep.	N6128	treble	feedback	3a, 4, 5, 6a	0.297158	0.844423	0.4577613	0.1432916	0.4278285	0.3561362	0.5107408

Alignment scores 8. IOR treble alignment: feedback/no feedback.

GROUP	SUBJECT	VOICE	CONDITION	BLOCKS	Min	Max	Mean	Stand. dev	Median	25 prcntil	75 prcntil
Improvising	N3851	treble	no feedback	1,2	0.3125	0.821531	0.5626924	0.1720174	0.56213	0.411133	0.735034
Improvising	N3933	treble	no feedback	1,2	0.399405	0.774082	0.5940061	0.1116224	0.595089	0.499731	0.671471
Improvising	N3938	treble	no feedback	1,2	0.183808	0.853002	0.4969852	0.2175903	0.419957	0.312112	0.695509
Improvising	N3974	treble	no feedback	1,2	0.167805	0.825885	0.5731394	0.1929279	0.5746745	0.4526487	0.7159933
Improvising	N4223	treble	no feedback	1,2	0.220969	0.796496	0.5303411	0.236926	0.621826	0.274297	0.74771
Improvising	N4229	treble	no feedback	1,2	0.165711	0.789875	0.5051093	0.1844934	0.512876	0.38761	0.592833
Improvising	N4258	treble	no feedback	1,2	0.1	0.786225	0.4024495	0.2198223	0.372283	0.224847	0.605175
Improvising	N4486	treble	no feedback	1,2	0.111111	0.702707	0.4179477	0.1770625	0.412017	0.344173	0.502169
Improvising	N4549	treble	no feedback	1,2	0.287136	0.860371	0.5976452	0.186957	0.677421	0.442719	0.738985
Improvising	N4774	treble	no feedback	1,2	0.301298	0.785091	0.5121794	0.1514482	0.520464	0.34573	0.628969
Improvising	N4869	treble	no feedback	1,2	0.429792	0.900558	0.6086987	0.1563606	0.596646	0.467814	0.682836
Improvising	N5692	treble	no feedback	1,2	0.227357	0.876084	0.4596631	0.1735439	0.377897	0.362198	0.56105
Score-dep.	N4429	treble	no feedback	1,2	0.182826	0.843865	0.3887297	0.1945463	0.353488	0.234209	0.536909
Score-dep.	N4517	treble	no feedback	1,2	0.200374	0.677532	0.4200151	0.1624161	0.428501	0.278342	0.595695
Score-dep.	N4588	treble	no feedback	1,2	0.273528	0.573209	0.4081923	0.1046994	0.42703	0.3105195	0.4976575
Score-dep.	N4615	treble	no feedback	1,2	0.216372	0.863107	0.5755065	0.215657	0.561917	0.381125	0.756875
Score-dep.	N4657	treble	no feedback	1,2	0.1	0.569799	0.2754686	0.1437641	0.254913	0.142857	0.3821022
Score-dep.	N5064	treble	no feedback	1,2	0.2322	0.67163	0.487939	0.1454337	0.504616	0.355048	0.628531
Score-dep.	N5480	treble	no feedback	1,2	0.329498	0.782574	0.5267097	0.1318584	0.516822	0.429519	0.648817
Score-dep.	N5484	treble	no feedback	1,2	0.111111	0.784548	0.4520026	0.2054495	0.360164	0.341377	0.698351
Score-dep.	N5783	treble	no feedback	1,2	0.270056	0.674897	0.4606256	0.1527249	0.380047	0.3335325	0.6134495
Score-dep.	N6128	treble	no feedback	1,2	0.295255	0.746195	0.4845222	0.1203298	0.508906	0.429559	0.524474
