# Supplementary material for: Behavioral Quantification of Audiomotor Transformations in Improvising and Score-Dependent Musicians
Source: PLoS One. 2016 Nov 11;11(11):e0166033. doi: 10.1371/journal.pone.0166033 (PMC5105996; doi:10.1371/journal.pone.0166033)
Supplement: S1 Alignment Scores — (ZIP) [file pone.0166033.s001.zip › Alignment_scores_9.pdf]

Alignment scores 9. Exact pitch bass alignment: feedback/no feedback.

GROUP	SUBJECT	VOICE	CONDITION	BLOCKS	Min	Max	Mean	Stand. dev	Median	25 prcntil	75 prcntil
Improvising	N3851	bass	feedback	3a, 4, 5, 6a	-0.25	1	0.2333562	0.3767438	0.2	-0.096875	0.5397725
Improvising	N3933	bass	feedback	3a, 4, 5, 6a	-0.295455	1	0.2955901	0.4075147	0.288961	-0.045455	0.5653413
Improvising	N3938	bass	feedback	3a, 4, 5, 6a	-0.642857	1	0.021464	0.4060889	0	-0.3	0.285714
Improvising	N3974	bass	feedback	3a, 4, 5, 6a	-0.571429	1	0.0530966	0.4465593	-0.05	-0.166667	0.275
Improvising	N4223	bass	feedback	3a, 4, 5, 6a	-0.277778	0.7	0.2254123	0.3149421	0.218254	-0.072917	0.4625
Improvising	N4229	bass	feedback	3a, 4, 5, 6a	0.0416667	1	0.5439763	0.3221693	0.472222	0.3641828	0.897727
Improvising	N4258	bass	feedback	3a, 4, 5, 6a	-0.375	0.4	-0.082167	0.196314	-0.102679	-0.179688	-0.03125
Improvising	N4486	bass	feedback	3a, 4, 5, 6a	-0.277778	1	0.024458	0.3307044	-0.069444	-0.209821	0.15
Improvising	N4549	bass	feedback	3a, 4, 5, 6a	-0.444444	0.35	-0.057588	0.2347354	-0.09127	-0.254596	0.096875
Improvising	N4774	bass	feedback	3a, 4, 5, 6a	-0.3	1	0.2769941	0.3760571	0.2222225	0.0738889	0.4375003
Improvising	N4869	bass	feedback	3a, 4, 5, 6a	-0.5	1	0.0326137	0.4488859	0.0052084	-0.411111	0.2775975
Improvising	N5692	bass	feedback	3a, 4, 5, 6a	-0.357143	1	0.4506404	0.4996329	0.5634615	-0.070833	0.8875
Score-dep.	N4429	bass	feedback	3a, 4, 5, 6a	-0.423077	1	-0.074055	0.3535538	-0.139611	-0.30815	0.0357143
Score-dep.	N4517	bass	feedback	3a, 4, 5, 6a	-0.4	0.714286	0.0896217	0.3332532	0.1651785	-0.134503	0.206731
Score-dep.	N4588	bass	feedback	3a, 4, 5, 6a	-0.269231	0.785714	0.0445651	0.2802985	0.0093985	-0.178572	0.214286
Score-dep.	N4615	bass	feedback	3a, 4, 5, 6a	-0.166667	1	0.306572	0.3466449	0.125	0.0881579	0.599359
Score-dep.	N4657	bass	feedback	3a, 4, 5, 6a	-0.5625	0.625	-0.143368	0.26255	-0.171123	-0.25	-0.11875
Score-dep.	N5064	bass	feedback	3a, 4, 5, 6a	-0.272727	0.444444	0.0041032	0.2265479	-0.031731	-0.179688	0.1217533
Score-dep.	N5480	bass	feedback	3a, 4, 5, 6a	-0.125	1	0.6190736	0.4232301	0.763393	0.4065129	1
Score-dep.	N5484	bass	feedback	3a, 4, 5, 6a	-0.4375	0.4	-0.22785	0.2330835	-0.277778	-0.401786	-0.131868
Score-dep.	N5783	bass	feedback	3a, 4, 5, 6a	-0.25	0.375	-0.009292	0.1512772	-0.019231	-0.099432	0.0572917
Score-dep.	N6128	bass	feedback	3a, 4, 5, 6a	-0.277778	0.625	-0.020564	0.2749577	-0.08125	-0.192096	0

Alignment scores 9. Exact pitch bass alignment: feedback/no feedback.

GROUP	SUBJECT	VOICE	CONDITION	BLOCKS	Min	Max	Mean	Stand. dev	Median	25 prcntil	75 prcntil
Improvising	N3851	bass	no feedback	1,2	-0.4375	0.666667	0.1985111	0.369045	0.227273	0	0.5
Improvising	N3933	bass	no feedback	1,2	-0.3	1	0.2416507	0.3377202	0.214286	0.0454545	0.4
Improvising	N3938	bass	no feedback	1,2	-0.409091	1	0.0948118	0.4982184	-0.041667	-0.277778	0.375
Improvising	N3974	bass	no feedback	1,2	-0.3125	1	0.2656945	0.4520946	0.1833335	-0.093056	0.625
Improvising	N4223	bass	no feedback	1,2	-0.388889	1	0.0134986	0.3723569	0	-0.277778	0.0833333
Improvising	N4229	bass	no feedback	1,2	-0.3	1	0.3161255	0.4470896	0.25	-0.111111	0.666667
Improvising	N4258	bass	no feedback	1,2	-0.375	0.7	-0.011959	0.3200566	0	-0.3125	0.2
Improvising	N4486	bass	no feedback	1,2	-0.333333	0.5	-0.040657	0.2292383	-0.090909	-0.158334	0
Improvising	N4549	bass	no feedback	1,2	-0.35	0.625	0.0266381	0.3499587	-0.041667	-0.3125	0.375
Improvising	N4774	bass	no feedback	1,2	-0.277778	1	0.1884382	0.3771483	0.0625	0.0454545	0.5
Improvising	N4869	bass	no feedback	1,2	-0.375	0.55	0.0411125	0.2904291	0	-0.125	0.25
Improvising	N5692	bass	no feedback	1,2	-0.1875	1	0.6277681	0.3639544	0.666667	0.384615	1
Score-dep.	N4429	bass	no feedback	1,2	-0.333333	1	-0.028571	0.3721504	-0.125	-0.214286	0.0416667
Score-dep.	N4517	bass	no feedback	1,2	-0.285714	0.7	0.1168471	0.3082428	0.111111	-0.1875	0.357143
Score-dep.	N4588	bass	no feedback	1,2	-0.333333	0.111111	-0.118276	0.1660458	-0.173611	-0.243056	0.0488636
Score-dep.	N4615	bass	no feedback	1,2	-0.333333	1	0.2564755	0.3801705	0.285714	-0.125	0.428571
Score-dep.	N4657	bass	no feedback	1,2	-0.5	0.625	-0.026858	0.3836022	-0.125	-0.375	0.15
Score-dep.	N5064	bass	no feedback	1,2	-0.375	1	0.095202	0.3929042	0	-0.166667	0.35
Score-dep.	N5480	bass	no feedback	1,2	-0.333333	1	0.3398099	0.4400263	0.382353	-0.25	0.625
Score-dep.	N5484	bass	no feedback	1,2	-0.375	0.25	-0.214286	0.1899348	-0.285714	-0.322917	-0.154762
Score-dep.	N5783	bass	no feedback	1,2	-0.428571	0.5	-0.035853	0.2815022	-0.025	-0.328125	0.1354168
Score-dep.	N6128	bass	no feedback	1,2	-0.3125	0.625	0.0396826	0.3438977	0.0555556	-0.285714	0.416667
