# Supplementary material for: Behavioral Quantification of Audiomotor Transformations in Improvising and Score-Dependent Musicians
Source: PLoS One. 2016 Nov 11;11(11):e0166033. doi: 10.1371/journal.pone.0166033 (PMC5105996; doi:10.1371/journal.pone.0166033)
Supplement: S1 Appendix — (PDF) [file pone.0166033.s002.pdf]

# S1 Appendix. Alignment.

The similarity of the aural model and the performance of the participant was determined by the construction of an alignment. This approach has often been used in musicology, especially in folk song research where it has been used to study the variability of melodies in oral transmission. Wiora provides many manual alignments of related folk song melodies [27]. One of Wiora's alignments is shown in Figure 1 which shows how Wiora believed the two melodies were related:

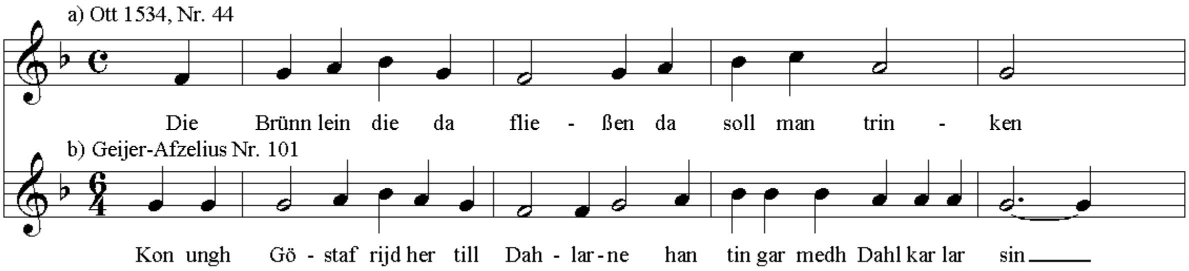


**Figure 1: Example of folk-song alignment as published by Wiora [27], (p. 161).**

Mongeau and Sankoff [28] applied an alignment algorithm to study the relation between theme and variations in Mozart's variations on 'Ah! vous dirai-je maman'. For algorithmic alignment, the procedure to construct an alignment is explicitly and exhaustively defined, such that it can be executed by a machine. There are many advantages: it is objective, replicable, and very quick. An important implication is that no implicit musical knowledge can be used, as was the case with, for example, the alignments of Wiora, which are handcrafted. All steps to construct an alignment need to be explicitly defined.

Consider the two melodies that are shown in Figure 2. They show similarities and differences. One possible alignment of these two melodies is depicted in Figure 3. The notes that correspond with each other according to this alignment are printed directly below each other.

Some notes do not have a corresponding note in the other melody. These notes are aligned with gaps. One could easily see that the extent to which it is possible to construct an alignment is related to the sequential similarity of two melodies. To quantify this ‘extent’ we assign numerical scores to each of the elements of the alignment.

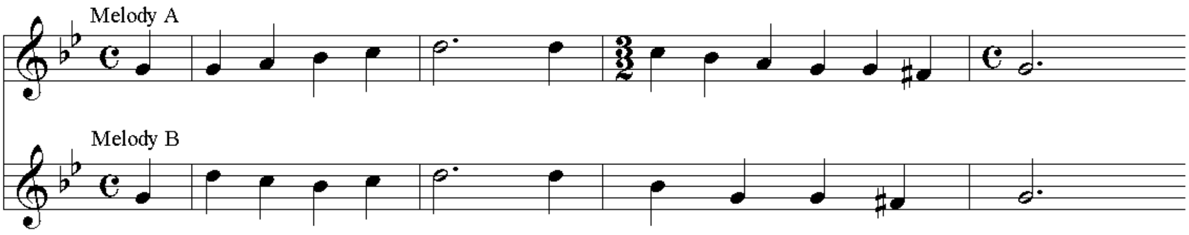


**Figure 2: Two melodies.**

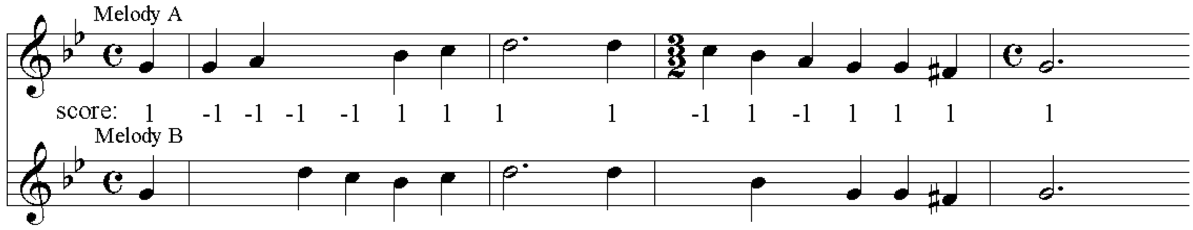


**Figure 3: One possible alignment of melodies A and B with a total score of 4.**

In the example in Figure 3, a very simple scoring scheme is used, which assigns score 1 to an alignment of two matching notes, and score -1 to the insertion of a gap. If we sum the individual scores, we end up with a total score of 4 for the alignment. Another possible alignment is shown in Figure 4. Here the distribution of gaps is different, and also non-matching notes are allowed to be aligned in measure two, be it with a negative score. The total score for this alignment is -2, which is lower than the alignment in Figure 3, implying that the first alignment is better, given the scoring scheme that we used. In general, with a scoring

scheme that assigns higher scores to matching notes and lower scores to mismatching notes and gaps, the more similar the two melodies are, the higher the total score of the alignment.

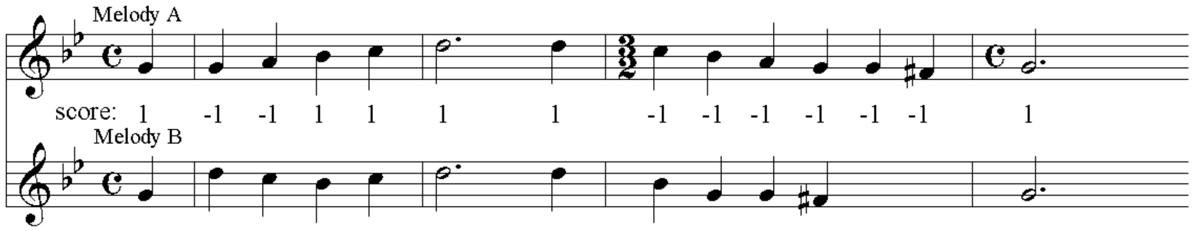


**Figure 4: Another possible alignment of melodies A and B with a total sum of -2.**

Consider two sequences of symbols x : $x_1$…...,$x_i$……, $x_n$, and y : $y_1$…., $y_j$…..,$y_m$, as depicted in Figure 5. In constructing an alignment of x and y, symbol $x_i$ can either be aligned with a symbol from sequence y or with a gap. Obviously, there are many ways to align two sequences since there are many possibilities to distribute gaps over the sequences.

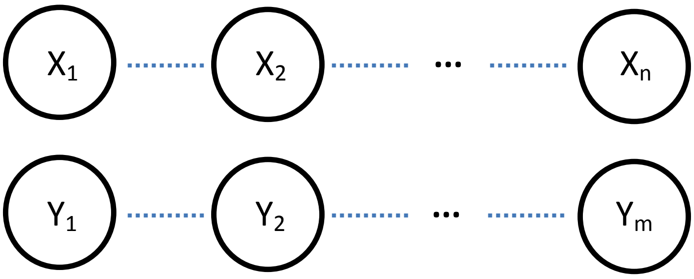


**Figure 5: Two sequences of symbols.**

As an example, Figure 6 shows just two randomly chosen possibilities of all conceivable alignments of sequences of lengths n = 7 and m = 6. In order to be able to compare alignments, each alignment of two sequences gets a score which expresses the similarity of the two sequences. This score is the sum of the scores of the alignments of the individual

symbols. In the first example, $y_1$ is aligned with a gap, so that receives the gap-score $\gamma$. $y_2$ is aligned with $x_1$ which receives the substitution score $S(x_1, y_2)$ and so on.

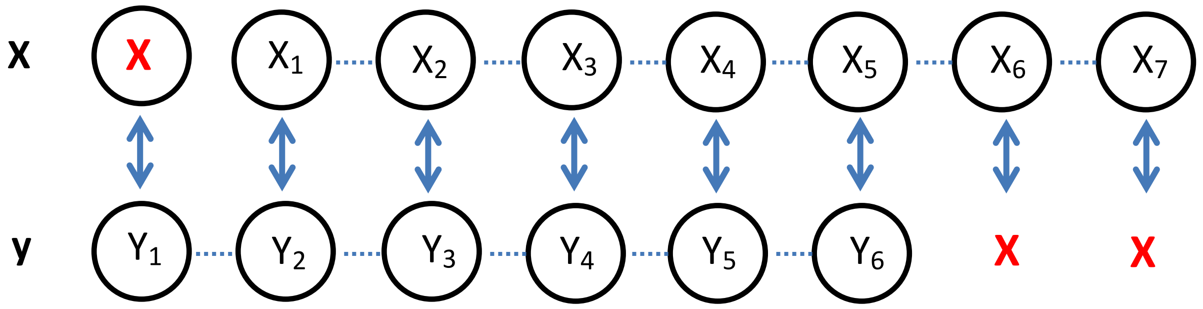


Score: $\gamma \quad + \quad S(x_1, y_2) + \ S(x_2, y_3) \ + S(x_3, y_4) + S(x_4, y_5) + S(x_5, y_6) \quad + \quad \gamma \quad + \quad \gamma$

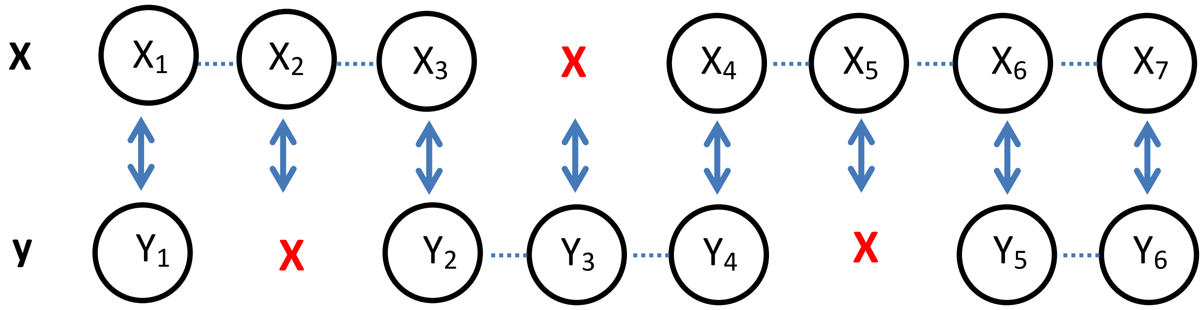


Score: $S(x_1, y_1) \quad + \quad \gamma \quad + \quad S(x_3, y_2) \quad + \ \gamma \ + \quad S(x_4, y_4) \quad + \quad \gamma \quad + \quad S(x_6, y_5) + (S(x_7, y_6)$

**Figure 6: Two possible alignments of sequences x and y with lengths n = 7 and m = 6.**

The aim of an alignment algorithm is to find the (or one of the) alignments(s) with the highest score. Since the solution space is quite large, generally a dynamic programming approach is taken to find the optimal alignment efficiently. In its simplest form, the optimal alignment and its score are found by filling a matrix D recursively according to:

$$D(i,j)=\max\begin{cases}D(i-1,j-1)+S(x_i,y_j)\\ D(i-1,j)+\gamma\\ D(i,j-1)+\gamma\end{cases}\quad, i\in[1,...n], j\in[1,...m]$$

in which $S(x_i,y_j)$ is a similarity measure for symbols and $\gamma$ is the (fixed) gap score. $D(0,0)=0$, $D(i,0)=i\gamma$, and $D(0,j)=j\gamma$. $D(i,j)$ contains the score of the optimal alignment up to $x_i$ and $y_j$, and therefore, $D(n,m)$ contains the score of the optimal alignment of the complete sequences. We can obtain the alignment itself by tracing back from $D(n,m)$ to $D(0,0)$; the algorithm has both time and space complexity $O(nm)$, which is quadratic. This algorithm is known as the Needleman-Wunsch algorithm [32].
