# Supplementary material for: Behavioral Quantification of Audiomotor Transformations in Improvising and Score-Dependent Musicians
Source: PLoS One. 2016 Nov 11;11(11):e0166033. doi: 10.1371/journal.pone.0166033 (PMC5105996; doi:10.1371/journal.pone.0166033)

S1 Transcriptions. Music excerpts.

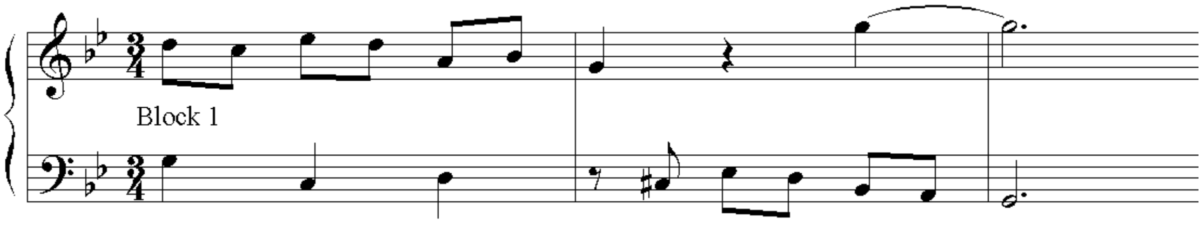

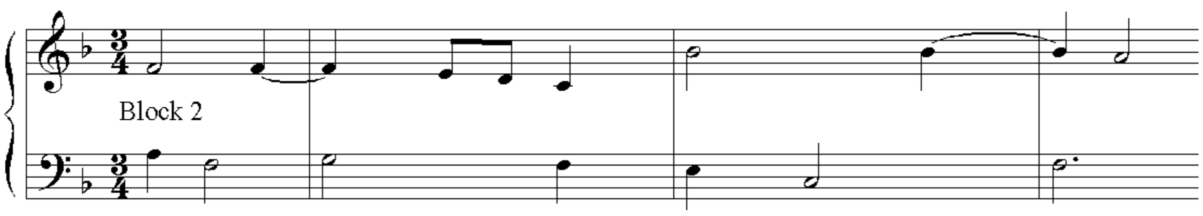

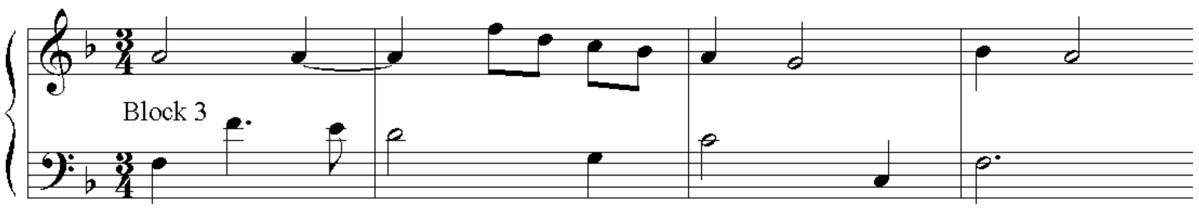

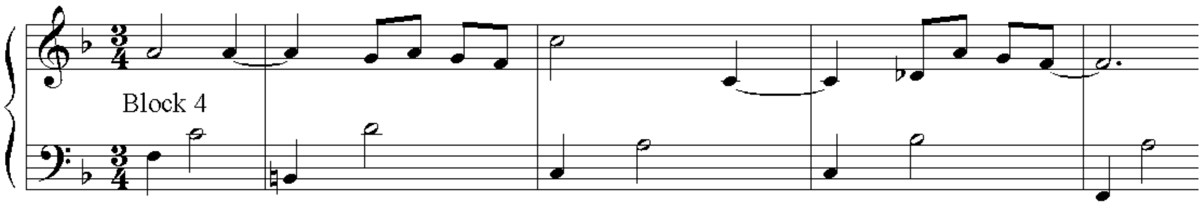

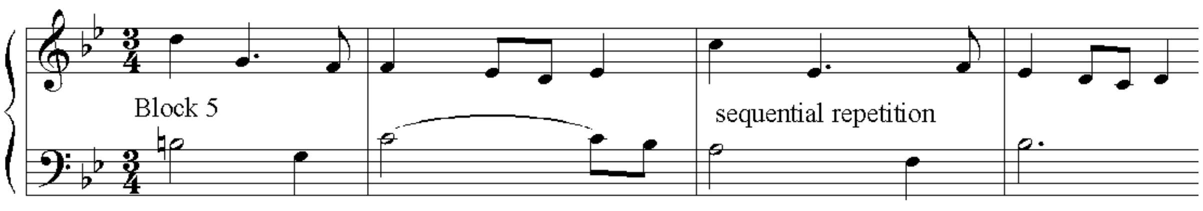

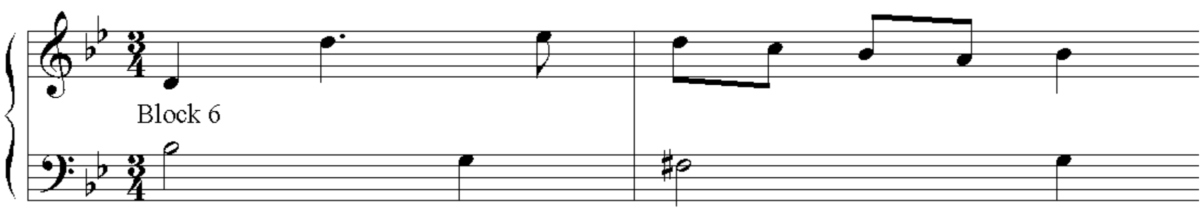

Supplement: S1 Transcriptions — (PDF) [file pone.0166033.s004.pdf]
